# Evidence-based core information for health communication of tobacco control: The effect of smoking on risks of female disease

**DOI:** 10.3389/fpubh.2022.986430

**Published:** 2022-10-18

**Authors:** Jin Liu, Yun-Yi Hao, Hui-Jia Mao, Xiang-Ju Sun, Xiao-Lu Huang, Chen-Xin Quan, Mei-Ling Cao, Shu-Ting Wei, Xue-Zheng Jin, Yi-Bo Wu

**Affiliations:** ^1^The Second Affiliated Hospital, China Medical University, Shenyang, China; ^2^School of Public Health, Shandong University, Jinan, China; ^3^School of Pharmaceutical Sciences, Jiangxi University of Traditional Chinese Medicine, Nanchang, China; ^4^The Fourth Affiliated Hospital, Harbin Medical University, Harbin, China; ^5^The Third Clinical Department, China Medical University, Shenyang, China; ^6^School for Policy Studies, University of Bristol, Bristol, United Kingdom; ^7^Affiliated Hospital of Integrated Traditional Chinese and Western Medicine, Nanjing University of Traditional Chinese Medicine, Nanjing, China; ^8^School of Basic Medical Sciences, Shandong University, Jinan, China; ^9^Department of Health Communication, Chinese Center for Health Education, Beijing, China; ^10^School of Public Health, Peking University, Beijing, China

**Keywords:** tobacco control, smoking, smoking cessation, female diseases, health communication, core information

## Abstract

**Objective:**

Cigarettes have become the the biggest killer of contemporary female's health and beauty. What kind of health information is suitable for the general public is an important issue to be discussed globally. The purpose of this study is to generate systematic, rigorous, public-demand-oriented and appropriate core information relevant to tobacco control based on the best available evidence, combined with audience preferences and pre-dissemination content review from multidisciplinary expertise in order to improve the effectiveness of health communication of tobacco control.

**Methods:**

Relevant systematic reviews meta-analysis that reported smoking on risks of female disease were identified by searching PubMed, Embase, the Cochrane Library, Web of Science, Clinical Trials.gov, and the International Clinical Trial Registry Platform. The Grading of Recommendations Assessment, Development and Evaluation (GRADE) process was applied to assess the evidence in order to make rigorous core information. The audience prevalence survey was conducted to ensure that core information was targeted and tailored. Finally, the expert assessment was used for a pre-dissemination content review and to evaluate whether the core information was appropriate or not.

**Results:**

The final core information consisted of eight parts concerning the effects of smoking and female cardiovascular disease, diabetes, rheumatoid arthritis, respiratory disease, digestive system disease, mental disease, non-pregnant female reproductive system disease, as well as pregnant women and their fetuses. A total of 35 items of core information suitable for dissemination was included and the quality of evidence, the degree of public demand and the outcome of pre-dissemination content review were reported.

**Conclusion:**

The core information related to female cardiovascular system diseases, as well as liver cancer and upper gastrointestinal cancer is the preferred content for health communication of tobacco control. The quality of evidence for core information related to pregnant women and their infants, as well as diseases of reproductive system, respiratory system, and diabetes needs to be improved to meet high public demand. The core information related to mental disease is more suitable for dissemination to patients with mental illness than to the general public. Besides, dissemination of core information should be individualized. Evidence-based Core Information for Health Communication of Tobacco Control would be helpful to provide evidence support for health communication related to tobacco control and enhance public health literacy for international communities that have high smoking prevalence and related disease burden.

## Introduction

The sheer number of dieases and deaths caused by tobacco underscores the importance of tobacco control as an urgent global health priority (1–5), especially for women, with their special physiological conditions which are different from males, the harm of smoking to them deserves more attention (6–14). The nicotine in tobacco can reduce the production of estrogen in women, which can lead to disorders in the body and the development of tumors (10, 11). And smoking can significantly reduce activated immune cells and lymphocytes, thereby reducing the immunity of the reproductive tract, increasing the risk of female gynecological diseases, and adversely affecting reproduction (12). The magnitude of change in trends in female smoking prevalence over the past 20 years signifies that female prevalence has not changed significantly since 2000 in most countries, or declined by < 10 percentage points (6, 7). Recently, a global smoking epidemic data shows that female smoking prevalence was 34–40% in Pacific Island countries (Nauru, Micronesia, Kiribati), 10–19% in several OECD (the Organization for Economic Co-operation and Development) countries (Canada, the USA, the UK, Australia and New Zealand) as well as many countries in Western Europe, and < 2–3% in several countries in Africa, as well as Tajikistan and Sri Lanka (6, 7).

Substantial global effort was devoted to curtailing the tobacco epidemic over the past two decade (15), including health communication for tobacco control which is an important part of tobacco control measures (e.g., tobacco pictorial health warnings, point-of-sale health communication campaigns, tobacco prevention education campaigns in university, media campaigns for tobacco control, interpersonal communication in tobacco control campaigns) (16–20), the pace of progress in reducing smoking prevalence has been with little success due to heterogeneous across development status, public literacy and age (21) as well as complicated evidence (7).

As demonstrated by GBD 2015 Tobacco Collaborators (21), countries with higher SDI (Socio-demographic Index) have higher prevalence of daily smoking and age patterns varied more by SDI. Female smoking prevalence typically peaked around age 25 years for high and high-middle SDI countries, while prevalence generally increased until age 60 years in low to middle SDI countries (21). This reminds us that tailored tobacco control health communication strategies (22–24) need to be adopted for different development status, public literacy and age pattern. Furthermore, health communication of tobacco control requires systematic, rigorous, targeted and appropriate evidence on smoking level. To meet this demand in the context of health communication, we propose “core information,” an unprecedented concept. Core information is condensed and suitable health information to be provided to health workers for dissemination to the public. Its development refers to the methods of evidence formation from WHO handbook, based not only on the best available evidence, but also on audience preferences and pre-dissemination content review from multidisciplinary expertise.

Therefore, the Evidence-based Core Information for Health Communication of Tobacco Control is formed in order to improve the effectiveness of health communication of tobacco control and subsequently reduced smoking prevalence, risks of related disease. It will also be helpful to enhance public health literacy for international communities that have high smoking prevalence and related disease burden.

## Methods

### Preliminary research basis for core information

**Figure 1:**
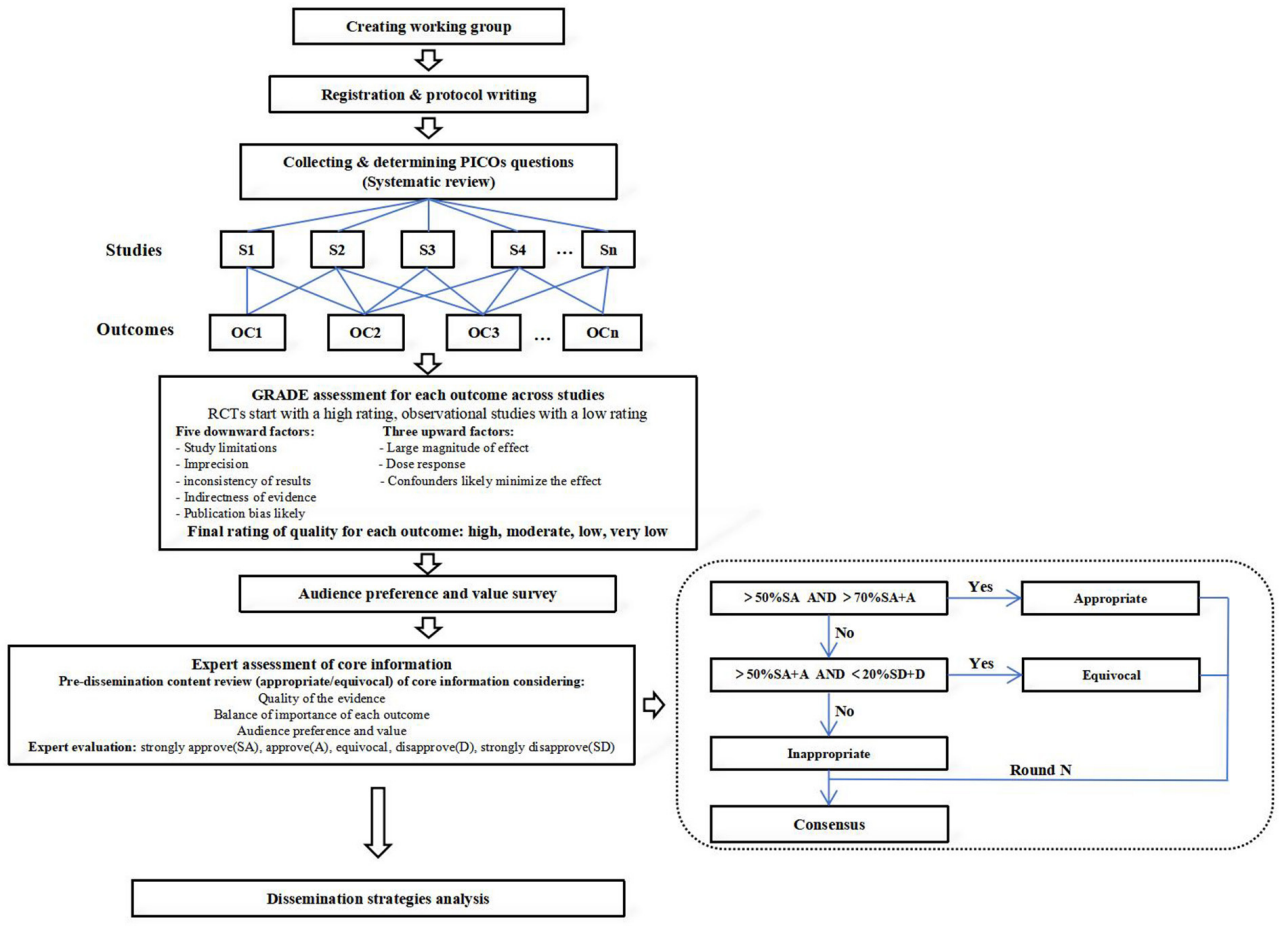
The technical route for development of Evidence-based Core Information for Health Communication of Tobacco Control.

This study was supposed to develop according to the methods of evidence formation from WHO handbook (25). The study followed the latest definition of evidence formation of Institute of Medicine(IOM) (26, 27) and conformed to the requirements for evidence in Reporting Items 2.0 for Practice Guidelines in Healthcare(RIGHT) (28), the RIGHT statement (29) and Appraisal Of Guidelines For Research & EvaluationII(AGREE II) (30). The technical route for development of Evidence-based Core Information for Health Communication of Tobacco Control is shown in Figure 1.

This study was initiated by the Health Communication Working Committee of the Chinese Medical Doctor Association, with methodological support provided by the Drug Evaluation Center of Peking University School of Medicine, and academic support provided by China Smoking Control Association, Beijing Smoking Control Association and other units. And this project was registered on the International Practice Guidelines Registry Platform (http://www.guidelines-registry.org/). The registration number was IPGRP-2020CN072.

### Guideline evidence

#### Literature search and screening

PICOs (participants, female; interventions (equivalent to exposure), smoking; comparisons, smokers vs. non-smokers; outcomes, incidence and mortality of various diseases; study types, systematic reviews) questions (10) followed literature pre-retrieving, International guideline reference and consulting with clinicians, clinical pharmacists, nurses, behavioral epidemiologists and health communication workers. Then the search strategy was determined through the final PICOs questions. To ensure the comprehensiveness of the evidence, we searched PubMed, Embase, and Web of Science, which are recognized as authoritative medical databases worldwide. The Cochrane Library was searched for evidence-based medical literature. Considering unpublished studies and their study details, we also searched Clinical Trials.gov and the International Clinical Trial Registry Platform. The retrieval time was from database construction to February 2021. The search formula consisted of keywords related to smoking and related diseases. Taking smoking and cardiovascular disease in women as an example, the search terms included: (cigarette OR cigarettes OR tobacco OR smoking OR smoking) AND (hypertension OR blood pressure) OR cancer OR (CVD OR cardiovascular disease OR coronary cardiovascular disease OR coronary heart disease). The language of the publication was Chinese or English and systematic reviews and meta-analysis were included. References and gray literature information (i.e., non-published literature, e.g., non-published government literature, dissertations, etc.) of the included literature were also manually searched and screened. The literature screening process was completed independently by two trained researchers. If there was a disagreement, a third researcher would join the discussion to reach a consensus.

#### GRADE accessment

The GRADE (Grade Recommendations Assessment, Development, and Evaluation) tool (31) was used to evaluate the quality of evidence in order to make rigorous core information (For specific implementation content, see Supplementary material 1). The evidence quality was divided into four levels: high, medium, low and very low (Table 1), considering five downward factors (study limitations, imprecision, inconsistency of results, indirectness of evidence and publication bias likely) and three upward factors (large magnitude of effect, dose effect and confounders likely minimize the effect) (Figure 1 and Supplementary material 1).

**Table 1:** Grade Recommendations Assessment, Development, and Evaluation (GRADE) system.

**Score**	**Grade**	**Definition**
2	High	Further research is very unlikely to change confidence in the estimate of effect.
1	Moderate	Further research is likely to have an important impact on confidence in the estimate of effect and may change the estimate.
−1	Low	Further research is very likely to have an important impact on confidencein in the estimate of effect and is likely to change the estimate.
−2	Very low	Any estimate of effect is very uncertain

#### Audience preference and value survey

The core information development group conducted an audience preference and value survey on the content of the core information (For specific implementation content, see Supplementary material 2). 670 participants was recruited, taking into account the distribution of age, gender, education level, occupation, smoking situation and chronic diseases in line with socio-demographic characteristics. Researchers of the development group explained the background and necessity of core information and discussed them with participants (32). Participants rated the core information using the Likert Level 5 scale: 5 for very necessary, 4 for necessary, 3 for equivocal, 2 for unnecessary, and 1 for very unnecessary. the mean ± standard deviation of each core information and the overall were calculate. The mean ± standard deviation of the overall was 3.10 ± 0.10, based on which, the degree of public demand was divided into four levels: low, medium, high and very high (Table 2). Finally, the results were formed and summarized and analyzed to provide reference for expert assessment group.

**Table 2:** The description of public demand for core information.

**Public demand**	**Description**
High	Mean value of core information ≥3.10+0.10
Relatively high	3.10 ≤ Mean value of core information < 3.10+0.10
Moderate	3.10-0.10 ≤ Mean value of core information < 3.10
Low	Mean value of core information was < 3.10-0.10

#### Expert assessment of core information

Expert assessment process: An expert assessment survey (For specific implementation content, see Supplementary material 3) was used for the pre-dissemination content review and to evaluate whether the core information was appropriate or not based on quality of evidence, audience preferences and values, balance of importance of each outcome, equity, feasibility, acceptability etc. After two rounds of evaluation and modification of core information, the final version of core information suitable for dissemination was reached (see **Table 5** in Supplementary material 3) (33).

The experts participating in this survey were distributed across the country, including 32 multidisciplinary experts whose professional level was high (see Table 2 in Supplementary material 3). They voted anonymously through the Chinese online ‘Questionnaire Star' platform. This process was monitored by the guideline development group. If the voting ratio of “5” was more than 50% and the voting ratio of “5” + “4” was more than 70%, the item was appropriate; if the voting ratio of “5” + “4” was more than 50% and the voting ratio of “2” + “1” was < 20%, the item was equivocal (Figure 1) (29). In the remaining cases, it was deemed inappropriate.

## Results

### Summary of core information

#### The correlation between smoking and risks of non-pregnant female reproductive system disease

Female reproductive system diseases mainly include gynecological inflammation, gynecological tumors, menstrual disorders and infertility, which bring both physical and mental effects to women and seriously reduce their quality of life. According to WHO global report on trends in prevalence of tobacco use 2000-2025, there were as many as 244 million women using tobacco in 2018 (30). It has been shown that cigarette smoke contains a variety of toxins that affect reproductive function to varying degrees (10), and smoking is therefore considered to be an important factor in the increased prevalence and mortality of female reproductive disorders (31–38)(For summary of evidence, see Supplementary material 1). Our study found that the degree of public was concentrated in “Moderate” and “Relatively High,” but the quality of evidence needed to be improved to meet the general public demand (Table 3). The core information in this field can be used as the content of tobacco control health communication. The specific summary of core information of female reproductive system disease is shown in Table 3.

**Table 3:** The correlation between smoking and risks of female reproductive system disease.

**Number**	**Core information**	**Evidence quality**	**Expert assessment**	**Public demand**
**7 The correlation between smoking and risks of non-pregnant female**				
**reproductive system disease**				
**7.1**	Smoking decreases the risk of non-pregnant female endometrial cancer	Moderate	Equivocal	Moderate
**7.2**	Smoking increases the risk of non-pregnant female cervical cancer	Very low	Equivocal	Moderate
**7.3**	Smoking increases the risk of non-pregnant female breast cancer	Low	Equivocal	Moderate
**7.4**	Smoking increases the risk of non-pregnant female serous or/and mucinous tumors of ovary	Very low	Equivocal	Moderate

#### Effects of smoking on pregnant women and their fetuses

Considering that pregnancy is a special period for women, pregnant women and fetuses are more susceptible to external influences during this period. Studies have shown that prenatal exposure to maternal smoking during pregnancy is harmful to mothers and fetuses (10, 39–44), including congenital heart disease, schizophrenia, attention deficit and hyperactivity disorder in the fetus as well as postpartum depression, coronary heart disease, abnormal pregnancy and all stages of reproduction in pregnant women. Our study revealed that the public had the greatest demand for core information in this field, but the quality of evidence needed to be improved (Table 4). The core information in this field can be used as the content of tobacco control health communication.

**Table 4:** Effects of smoking on pregnant women and their fetuses.

**Number**	**Core information**	**Evidence quality**	**Expert assessment**	**Public demand**
**8 Effects of smoking on pregnant women and their fetuses**				
**8.1**	Smoking can adversely affect the various stages of pregnancy in female reproduction (reproductive function, follicular development, steroid development, embryo transfer, endometrial receptivity, endometrial angiogenesis, uterine blood flow and myometrium)	Very low	Equivocal	High
**8.2**	Smoking increases the chance of abnormal pregnancy in pregnant women	Very low	Equivocal	High
**8.3**	Smoking increases the risk of postpartum depression in pregnant women	Low	Equivocal	Relatively high
**8.4**	Smoking increases the risk of fetal schizophrenia in pregnant women	Very low	Equivocal	High
**8.5**	Smoking increases the risk of fetal attention deficit and hyperactivity disorder in pregnant women	Moderate	Equivocal	High
**8.6**	Smoking increases the risk of fetal coronary heart disease in pregnant women	Very low	Appropriate	High

#### The correlation between smoking and risks of female respiratory disease

Of all risk factors, Smoking is the leading risk factor for chronic respiratory diseases, For women, the leading cause of smoking-attributable DALYs is COPD (21). Evidences found that female current smokers had with increasing age (especially beyond age 45 to 50 years in the pre and post menopausal periods) a significantly faster annual decline in FEV1% (a golden indicator of COPD) predicted than male current smokers (linear regression analysis, R^2^ = 0.56; *p* = 0.008), and this trend was evident even in female smokers who smoked only a modest amount of cigarettes (<15 g/day) (45). (For summary of evidence, see Supplementary material 1). Besides, smoking may also have a greater negative impact on lung growth in female than male during childhood and adolescence (46, 47). This suggests that the relationship between gender, age and changes in FEV1% may be U-shaped. The mechanisms responsible for the increased susceptibility of women than men to cigarette smoke can be summarized as: (1) smoking-inflammation pathway in the pre and post-menopausal periods (48, 49); (2) bronchial hyperresponsiveness in women compared to men (50–52); (3) hormonal status (53); (4) differences in lung development between females and males (54–56). Our study showed that the public demand for core information in this area was high, but the quality of evidence was low (Table 5). The dangers of smoking on the respiratory system are obvious, but evidence for women is still lacking. This is because women smoke less than men, so most surveys focus on male groups. Therefore, it is of great urgency to explore high-quality and sufficient evidence in this area to meet public health needs, and pay more attention to health education for adolescent and menopausal female. The specific summary of core information of female respiratory disease is shown in Table 5.

**Table 5:** The correlation between smoking and risks of female diabetes, rheumatoid arthritis and respiratory disease.

**Number**	**Core information**	**Evidence quality**	**Expert assessment**	**Public demand**
**2 The correlation between smoking and risks of female diabetes**				
**2.1**	Smoking increases the risk of female type 2 diabetes	Very low	Equivocal	Moderate
**3 The correlation between smoking and risks of female rheumatoid arthritis**				
**3.1**	Increased smoking increases the risk of female rheumatoid arthritis	Very low	Equivocal	Low
**4 The correlation between smoking and risks of female respiratory disease**				
**4.1**	Smoking increases the risk of female chronic obstructive pulmonary disease	Low	Appropriate	High

#### The correlation between smoking and risks of female cardiovascular disease

Several studies have shown that smoking is recognized as independent risk factors for cardiovascular diseases (21), which not only increases the oxidative stress, causing the oxidation of low-density lipoproteins (LDL), but also increases inflammation (57). It affects all stages of atherosclerosis, and eventually leads to cardiovascular diseases. In postmenopausal women, the estrogen, regarded as the guardian of blood vessels, is greatly reduced, which results in increased LDL and decreased high-density lipoproteins (HDL), thereby accelerating hardening of the arteries and leads to an increased risk of coronary heart disease, heart failure, sudden cardiac death, stroke and overall cardiovascular diseases (58–62) (For summary of evidence, see Supplementary material 1). Our research found that the quality of evidence, the degree of public demand and the results of expert assessments were relatively consistent and high in this area (Table 3). This is because cardiovascular diseases are closely related to people's lives and carry a high risk of death. Researches have found that the global mortality of cardiovascular diseases has increased by 12.5% over the past decade, and cardiovascular diseases currently account for about one-third of global deaths (63), so preventing cardiovascular diseases will greatly improve global health. This means that the core information in this field is the preferred content for health communication of tobacco control. Furthermore, It is worth noting that lifestyle and other factors are important control variables and should be considered in the health communication of tobacco control when discussing the impact of smoking on female cardiovascular disease. Smokers are more likely to have poor lifestyles like high-fat diet (increases triglyceride and cholesterol levels in the blood, causing hyperlipidemia and atherosclerosis), lack of exercise (leads to obesity and metabolic syndrome) and being sedentary (results in thrombosis), which jointly contribute to the development of cardiovascular diseases (64, 65). The specific summary of core information is shown in Table 6.

**Table 6:** The correlation between smoking and risks of female cardiovascular disease.

**Number**	**Core information**	**Evidence quality**	**Expert assessment**	**Public demand**
1 The correlation between smoking and risks of female cardiovascular disease				
1.1	Smoking increases the risk of female coronary heart disease	Moderate	Appropriate	High
1.2	Smoking increases the mortality of female coronary heart disease	Low	Appropriate	High
1.3	Smoking increases the mortality of female heart failure	Low	Appropriate	Relatively high
1.4	Smoking increases the risk of female sudden cardiac death	Moderate	Appropriate	High
1.5	Smoking increases the risk of female stroke	Moderate	Appropriate	Relatively high
1.6	Smoking increases the mortality of female stroke	Low	Appropriate	Relatively high
1.7	Smoking increases the risk of female overall cardiovascular disease	Moderate	Appropriate	High
1.8	Smoking increases the mortality of female overall cardiovascular disease	Moderate	Appropriate	High

#### The correlation between smoking and risks of female mental disease

Mental illness have a huge burden on health in the world today. According to statistics, the global burden of mental illness accounts for 32·4% of years lived with disability (YLDs) and 13·0% of disability-adjusted life-years (DALYs) (66). Smoking rate was high in patients with mental illness, and it was estimated that more than 200,000 of 520,000 people suffer from mental illness who died mainly from chronic diseases caused by smoking in the United States (67). Especially for women, changes in their sex hormone levels (for example, the “recession” of sex hormones in menopausal women aged 45–55 years old, causing a series of physiological changes, imbalances in Nervous system activity and reduced adaptability to the outside world), as well as social stereotypes and low inclusion of women, lead to a higher prevalence of depression in this group, resulting in high smoking rates. Besides, female smoking groups are more likely to be discriminated against and stigmatized in social life (68), and they themselves are more sensitive and vulnerable to social impact (such as more passive to be exposed to smoking, more susceptible to negative emotions with smoking addiction) (69), causing further damage to their mental health. Our study found that the quality of evidence, the degree of public demand and the results of expert assessments are relatively consistent and low in this area (Table 7). This is because the effects of smoking on psychiatric disorders are mainly indirect (66–68) and unclear (70, 71) (For summary of evidence, see Supplementary material 1). In most cases, it is the mental illness itself that leads to the action of smoking. Therefore, this result suggests to health communicators that core information in this field is less appropriate for dissemination to the general public compared with other core information, but is more suitable for patients with mental illnesses. The specific summary of core information of female mental disease is shown in Table 7.

**Table 7:** The correlation between smoking and risks of female mental disease.

**Number**	**Core information**	**Evidence quality**	**Expert assessment**	**Public demand**
6 The correlation between smoking and risks of female mental disease				
6.1	Smoking increases the risk of female Alzheimer's disease	Very low	Equivocal	Moderate
6.2	Smoking increases the risk of female all-cause dementia	Very low	Equivocal	Moderate

#### The correlation between smoking and risks of female digestive system disease

The chemicals in cigarette smoke including tobacco-specific nitrosamines, polycyclic aromatic hydrocarbons and aromatic amines play a major role in the development of digestive diseases by causing genetic mutations (72). Additionally, unhealthy diet like low fruit and vegetables intake (64), high fat eating (64), high salt consumption (73) and excessive alcohol ingestion (74, 75) (which itself has the effect of disease-promoting) and inappropriate daily habits like smoking after meals (which is harmful not only to the respiratory system, but also the digestive system) could be crucial reasons for an increased risk of digestive diseases, as these factors had synergistic effects with smoking. A total of 14 articles reported the effect of smoking on morbidity and mortality of female digestive diseases (76–89), including pancreatitis, pancreatic cancer, liver cancer, stomach cancer, upper gastrointestinal cancer, colon cancer, rectal cancer, diverticular disease, colitis (For summary of evidence, see Supplementary material 1). In this field, The quality of evidence and public demand for liver and upper gastrointestinal cancers were high, which might be related to the higher incidence of these cancers. For such diseases that are closely related to people's lives, these core information can be used as the first choice in the health communication of tobacco control, and the factors about the unhealthy diet and inappropriate daily habits mentioned above need to be paid enough attention to. The specific summary of core information of female digestive disease is shown in Table 8.

**Table 8:** The correlation between smoking and risks of female digestive system disease.

**Number**	**Core information**	**Evidence quality**	**Expert assessment**	**Public demand**
5 The correlation between smoking and risks of female digestive system disease				
5.1	Smoking increases the risk of female pancreatitis	Moderate	Equivocal	Moderate
5.2	Smoking increases the risk of female chronic pancreatitis	Moderate	Equivocal	Moderate
5.3	Smoking increases the risk of female pancreatic cancer	Low	Equivocal	Moderate
5.4	Smoking increases the mortality of female pancreatic cancer	Low	Equivocal	Moderate
5.5	Smoking increases the risk of female liver cancer	Low	Equivocal	Relatively high
5.6	Smoking increases the mortality of female liver cancer	Low	Equivocal	Relatively high
5.7	Smoking increases the risk of female gastric cancer	Low	Equivocal	Moderate
5.8	Smoking increases the risk of female upper gastrointestinal cancer	Moderate	Equivocal	Relatively high
5.9	Smoking increases the risk of female colon cancer	Low	Equivocal	Moderate
5.10	Smoking increases the risk of female rectal cancer	Low	Equivocal	Moderate
5.11	Smoking increases the risk of female diverticular disease	Low	Equivocal	Moderate
5.12	Smoking increases the risk of female colitis	Low	Equivocal	Moderate

#### The correlation between smoking and risks of female diabetes

Two studies have shown that smoking increases the risk of diabetes in women, with results of 1.42 (95%: 1.19, 1.69) and 1.33 (95%: 1.26–1.41) relative risks of diabetes for current smokers respectively (90, 91) (For summary of evidence, see Supplementary material 1). The prevalence of type 2 diabetes (T2D) has gradually increased over the past three decades and has become a major global public health challenge. Given the high prevalence of smoking in many countries and the increasing burden of diabetes worldwide, reducing tobacco use should be prioritized as an important public health strategy that may contribute to the prevention and control of diabetes (90). This was also in line with a moderate public demand for core information in this area, but the quality and quantity of evidence was low (Table 4). This suggests that high-quality and sufficient evidence is urgently needed in this field to meet public health needs. The specific summary of core information of female diabetes is shown in Table 5.

#### The correlation between smoking and risks of female rheumatoid arthritis

Rheumatoid arthritis (RA) is a major autoimmune disease and is typically characterized by chronic inflammation of the articulations and bone destruction (92). As the most common form of inflammatory arthritis, its worldwide prevalence is approximately 1%, with women at two to three times the risk of developing rheumatoid arthritis compared to men due to the effect of estrogen on the immune system. The cumulative adult prevalence is 3.6% for women and 1.7% for men (93). Two systematic reviews have shown that smoking increases the risks of rheumatoid arthritis in women (94, 95), and male smokers are at greater risk than female smoker (95–97) (For summary of evidence, see Supplementary material 1). But the results of different original articles were inconsistent. Whether smoking habits caused RA was not clear. Our study found that the quality of evidence, the degree of public demand and the results of expert assessments were relatively consistent and low in this area (Table 5). This result suggests to health communicators that core information in this field is less suitable for tobacco control health communication. The specific summary of core information of female rheumatoid arthritis is shown in Table 5.

### Dissemination analysis of core information

Dissemination of core information should be individualized. Based on audience preferences survey, we conducted a dissemination analysis of core information to guide health communicators how to spread the core information to audiences.

Different levels of education, gender and age had an effect on the degree of need for core information (Table 1 in Supplementary material 2). Among them, college students and undergraduates, women, and groups aged 26–45 were in greater demand. Most smokers were not only well educated but also aware of the dangers of smoking, however, they rarely received advice or help to quit (98). We suggest that middle-aged non-smoking women with higher education can be regarded as the main target for health communication workers to spread tobacco control core information. Because they can not only obtain more information about tobacco control for themselves and their families, but also play the role of supervision and persuasion.

Different occupations had different degrees of demand for core information, and the demand of medical workers was higher than that of non-medical workers, indicating that medical workers paid more attention to the impact of smoking on diseases (Table 1 in Supplementary material 2). Because of the particularity of their occupations, medical staff paid more attention to health-related information than other occupations, especially for the information needs of smoking hazards. Studies have shown that health education and health promotion can effectively improve medical staff's awareness of the dangers of smoking and their support for comprehensive smoking bans in medical and health institutions. This suggests that it is necessary to increase tobacco control education for medical personnel, and at the same time encourage and drive more medical personnel to contribute to medical education, scientific research, and international cooperation related to tobacco harm.

The public with chronic diseases had a higher demand for core information than the public without chronic diseases (Table 1 in Supplementary material 2). A large number of studies have shown that smoking is an important risk factor for the occurrence and death of various chronic non-communicable diseases, so people with these chronic diseases also paid more attention to the information on whether smoking control affected their health. In chronic disease management, the importance of smoking cessation-related health education was particularly important. It is recommended to increase the intensity of tobacco control health education among elderly patients with chronic diseases, which is of great significance for the effective management of chronic diseases, curbing the progress and adverse outcomes of the disease, and improving the quality of life of patients.

Compared with smokers, non-smokers had a higher degree of demand, indicating that non-smokers were more concerned about the information that smoking was harmful to health (Table 3 in Supplementary material 2). Our research also showed that the reason for the willingness to quit smoking among smokers was mainly “fear of getting sick.” Therefore, strengthening publicity and education about the harm of smoking can help smokers generate their willingness to quit (99).

Additionally, core information can be explained in a simple, science-based way that is more appropriate to the general public. This coincides with the importance of using visuals, telling real stories and using familiar language, as highlighted by the WHO Strategic Communication Framework for Effective Communications (100). Furthermore, attention should be paid to multi-culture group differences in health communication and tailored and targeted health communication strategies (22–24) are adopted for different groups. And different population groups based on the characteristics of the recipients (education, age and occupation) in the same culture context also need diversified communications to ensure effectiveness (101–103). A WHO scoping review noted that arts-based approaches are particularly useful in programming for multicultural groups and for building trust around sensitive health topics (104).

## Discussion

This study focused on women's health, with rigorous, public-demand-oriented and appropriate core information covering eight parts and proposing 35 core information to maximize coverage of the dangers of smoking on all systems in females and to provide guidance on the health communication for tobacco control. Of all the core information, the core information related to female cardiovascular system diseases, as well as liver cancer and upper gastrointestinal cancer is the preferred content for health communication of tobacco control. The quality of evidence for core information related to pregnant women and their infants, as well as diseases of reproductive system, respiratory system, and diabetes needs to be improved to meet high public demand. The core information related to mental disease is more suitable for dissemination to patients with mental illness than to the general public. Besides, dissemination of core information should be individualized.

Advantages of this study are as follows. Initially, it is the first systematic and comprehensive evidence-based core information for health communication in the field of tobacco control, which which will be of great significance to improve the reliability of evidence. In the past, tobacco control in health communication was primarily composed of the media communication channels (105, 106), whose reliability was often influenced by some large stakeholders, which led to frequent rumors in this field (7). Secondly, the formation of core information took into account the needs of the public with a reasonable distribution of age, gender, education level, occupation, smoking status and chronic disease status according to sociodemographic characteristics during the survey. This can ensure that the core information keeps “the common touch.” Thirdly, the expert assessment pre-dissemination content expert review from multidisciplinary expertise (29) was used and factors such as the quality of evidence, the degree of public demand, balance of importance of each evidence, equity, feasibility, acceptability were considered comprehensively so that core information is not only scientifically correct, but also suitable for dissemination.

However, this study has certain limitations. First, available evidences supporting this core information were actually weak due to ethical concerns (smoking is a detrimental factor and not suitable as an intervention), which contributed to a lack of large-scale randomized controlled trials (RCTs) and then led to scarcity of high quality evidence. Secondly, the public prevalence survey and expert assessment were mainly confined to the national conditions of China, which resulted in limitations in the scope of application. Thirdly, given that the overall number of smoking women was still less than that of men, related to specific diseases like rheumatoid arthritis, might be deviated from the objective reality.

For future perspectives, there are several important areas that need to be improved:

First, smoking and passive smoking are recognized as one of the main causes of cardiovascular diseases, and they are also independent risk factors for such diseases (21). Evidences show that the relationship between smoking and cardiovascular disease has received widespread attention and is of relatively high quality, recommendation and public need. Future investigations still require high-quality researches in this area.

Second, the relationship between smoking and respiratory disease is well known (21), as the respiratory system is the first system to be exposed to smoke and is susceptible to oxidative damage, resulting in chronic inflammatory responses and airway remodeling and, in severe cases, cancerous lesions. However, the available research lacks separate data on women and extensive empirical epidemiological evidence. Future development in this areas is of great value to assist tobacco control in women.

Third, future investigations on effects of smoking on pregnant women and their fetuses are of great importance to assist tobacco control in women due to greater susceptibility to tobacco among pregnant women and fetuses during pregnancy. At the same time, researches on the harm of second-hand smoke (in a passive way) to pregnant women and their fetuses are also worthy of attention. It suggests that future researches can form core information about the effects of passive smoking on pregnant women and their fetuses.

Fourth, the current study focuses less attention on the effect of smoking on female mental diseases which is a huge burden on health in the world today (66, 67). As large-scale, prospective studies are actually lacking in this areas, further studies are required for more convincing evidence.

Fifth, core information concerning smoking on female rheumatoid arthritis was controversial and with low evidence and public need and weak recommendation. Future investigation in this field need to be more comprehensive, accurate and rigorous.

Sixth, tobacco control health communication strategies of core information of this guideline are also needed for more tailored and targeted for different development status, public literacy and age pattern.

Finally, future development of evidence-based core information of tobacco control for health communication can focus on the effectiveness of positive interventions on tobacco control, such as mobile health interventions; and include more high-quality and large-scale RCTs.

## Data availability statement

The original contributions presented in the study are included in the article/Supplementary material, further inquiries can be directed to the corresponding authors.

## Author contributions

Draft writing: JL, Y-YH, X-LH, Y-BW, and X-ZJ. Draft revising and reviewing: Y-BW and X-ZJ. Conception and design: Y-BW, X-ZJ, JL, and C-XQ. Guideline registration and protocol writing: JL, C-XQ, and S-TW. Collecting and determining PICOs questions: JL, Y-YH, H-JM, M-LC, and S-TW. GRADE assessment: JL and Y-YH. Audience value survey: X-JS and JL. Delphi expert consensus survey: JL, H-JM, Y-YH, Y-BW, and X-ZJ. All authors contributed to the article and approved the submitted version.

## Funding

This work was supported by the Bloomberg Initiative to Reduce Tobacco Use(CHINA-25-07).

## Conflict of interest

The authors declare that the research was conducted in the absence of any commercial or financial relationships that could be construed as a potential conflict of interest.

## Publisher's note

All claims expressed in this article are solely those of the authors and do not necessarily represent those of their affiliated organizations, or those of the publisher, the editors and the reviewers. Any product that may be evaluated in this article, or claim that may be made by its manufacturer, is not guaranteed or endorsed by the publisher.
