# Supplementary material for: Evidence-based core information for health communication of tobacco control: The effect of smoking on risks of female disease
Source: Front Public Health. 2022 Oct 18;10:986430. doi: 10.3389/fpubh.2022.986430 (PMC9623329; doi:10.3389/fpubh.2022.986430)
Supplement: Supplementary file 1 [file Data_Sheet_1.docx]

**Supplementary 1. Table 1 GRADE evidence profile: smoking and female cardiovascular disease**

| **Summary of findings** | | | | | | | **Quality assessment** | | | | | | |
| --- | --- | --- | --- | --- | --- | --- | --- | --- | --- | --- | --- | --- | --- |
| **Studies** | **Exposure** | **Case control** | **Cohort** | **Events/**  **Total** | **Relative risk (95% CI)** | **Risk difference (95% CI)** | **Limitations^a^** | **Inconsistency^b^** | **Indirectness^c^** | **Imprecision^d^** | **Publication bias^e^** | **Upward factors^f^** | **Quality** |
| **1.1 Smoking increases the risk of female coronary heart disease** | | | | | | | | | | | | | |
| Hackshaw 2018 | Smoking 1 per day | 0 | 18 | 29870/  2.34millio | RR=1.57 (1.29-1.91) | Significant | No serious limitations | Serious inconsistency -1 | No serious indirectness | No serious imprecision | Undetected | Large effect+1  Dose response+1 | +++  Moderate |
|  | Smoking 5 per day |  |  |  | RR=1.76 (1.46-2.13) |  |  |  |  |  |  |  |  |
|  | Smoking 20 per day |  |  |  | RR=2.84 (2.21-3.64) |  |  |  |  |  |  |  |  |
| **1.2 Smoking increases the mortality of female coronary heart disease** | | | | | | | | | | | | | |
| Woodward 2005 | Current smoking | 0 | 40 | 3010/  562338 | HR=1.73  (1,44-1,7) | Significant | No serious limitations | Serious inconsistency -1 | No serious indirectness | No serious imprecision | Undetected | Dose response+1 | ++  Low |
| **1.3 Smoking increases the mortality of female heart failure** | | | | | | | | | | | | | |
| Huxley 2014 | Current smoking | 0 | 21 | 579/543694 | HR=1.39  (1.02-1.89) | Significant | No serious limitations | No serious inconsistency | No serious indirectness | No serious imprecision | Undetected | / | ++  Low |
| **1.4 Smoking increases the risk of female sudden cardiac death** | | | | | | | | | | | | | |
| Aune 2018 | Current smoking | 0 | 12 | 1055/  138273 | RR=2.44 (1.80-3.31) | Significant | No serious limitations | No serious inconsistency | No serious indirectness | No serious imprecision | Undetected | Large effect+1 | +++ Moderate |
|  | Former smoking |  |  |  | RR=1.40 (1.10-1.79) |  |  |  |  |  |  |  |  |

| **1.5 Smoking increases the risk of female stroke** | | | | | | | | | | | | | |
| --- | --- | --- | --- | --- | --- | --- | --- | --- | --- | --- | --- | --- | --- |
| Hackshaw 2018 | Smoking 1 per day | 0 | 10 | 60520/  3.59million | RR=1.31 (1.13-1.52) | Significant | No serious limitations | Serious inconsistency -1 | No serious indirectness | No serious imprecision | Undetected | Large effect+1  Dose response+1 | +++ Moderate |
|  | Smoking 5 per day |  |  |  | RR=1.44 (1.22-1.70) |  |  |  |  |  |  |  |  |
|  | Smoking 20 per day |  |  |  | RR=2.16  (1.69-2.75) |  |  |  |  |  |  |  |  |
| Peters 2013 | smoking | 0 | 81 | 42401/  3980359 | RR=1,83  (1.58-2.12) | Significant | No serious limitations | No serious inconsistency | No serious indirectness | No serious imprecision | Undetected | / | ++  Low |
| **1.6 Smoking increases the mortality of female stroke** | | | | | | | | | | | | | |
| Woodward 2005 | smoking | 0 | 40 | 3131/562338 | HR-1.42(1.26-1.62) | Significant | No serious limitations | No serious inconsistency | No serious indirectness | No serious imprecision | Undetected | / | ++  Low |
| **1.7 Smoking increases the risk of female overall cardiovascular disease** | | | | | | | | | | | | | |
| Hackshaw 2018 | The number of cigarettes smoked per day | 0 | 4 | 36525/  1.00million | RR=1.65(1.13-2.40)  RR=1.74(1.30-2.34) | Significant | No serious limitations | Serious inconsistency -1 | No serious indirectness | No serious imprecision | Undetected | / | +++ Moderate |
| **1.8 Smoking increases the mortality of female overall cardiovascular disease** | | | | | | | | | | | | | |
| Hackshaw 2018 | The number of cigarettes smoked per day | 0 | 4 | 36525/  1.00million | RR=1.65(1.13-2.40)  RR=1.74(1.30-2.34) | Significant | No serious limitations | Serious inconsistency -1 | No serious indirectness | No serious imprecision | Undetected | / | +++ Moderate |

**a:**1. Failure to develop and use reasonable inclusion criteria: under- or over-matching in case-control studies, selection of exposed and non-exposed groups from different populations in cohort studies; 2. Weaknesses in both exposure and outcome measures: recall bias in case-control studies, differences in outcome monitoring between exposed and non-exposed groups in cohort studies; 3. Failure to adequately control for confounding: not accurately measuring all known prognostic factors, not matching for prognostic factors, and/or not adjusting for statistical analysis; 4. Incomplete follow-up.

**b:** When I2>75%, the quality of evidence is downgraded due to inconsistency.

**c:** 1. Differences in population; 2. Differences in exposure factors; 3. Differences in outcome measures (surrogate outcomes).

**d:** Uncertainty is downgraded when the overall effect size confidence interval includes 1.0.

**e:** The symmetry of the funnel plot can reflect its publication bias. Egger's P value: P > 0.05: severe -1; P > 0.05: undetected 0.

**f:** Upward factors include large effecte, dose response and reasonable confounding bias.

**Supplementary 1. Table 2 GRADE evidence profile: female diabetes**

| **Summary of findings Quality assessment** | | | | | | | | | | | | | |
| --- | --- | --- | --- | --- | --- | --- | --- | --- | --- | --- | --- | --- | --- |
| **Studies** | **Exposure** | **Case control** | **Cohort** | **Events/Total** | **Relative risk (95% CI)** | **Risk difference (95% CI)** | **Limitations** | **Inconsistency** | **Indirectness** | **Imprecision** | **Publication bias** | **Upward factors** | **Quality** |
| **2.1 Smoking increases the risk of female type 2 diabetes** | | | | | | | | | | | | | |
| Pan 2015 | Current and former smoking | 0 | 88 | ?/5853952 | RR=1.33(1.23-1.41)  RR=1.12(1.20-1.95) | Significant | No serious limitations | Serious inconsistency -1 | No serious indirectness | No serious imprecision | Undetected | / | +  very low |
| Akter 2017 | Current smoking | 0 | 19 | 16383/  343573 | RR=1.42(1.19-1.69) | Significant | No serious limitations | No serious inconsistency | No serious indirectness | No serious imprecision | Serious publication bias -1 | / | +  very low |

**Supplementary 1. Table 3 GRADE evidence profile: female rheumatoid arthritis**

| **Summary of findings Quality assessment** | | | | | | | | | | | | | |
| --- | --- | --- | --- | --- | --- | --- | --- | --- | --- | --- | --- | --- | --- |
| **Studies** | **Exposure** | **Case control** | **Cohort** | **Events/Total** | **Relative risk(95% CI)** | **Risk difference(95% CI)** | **Limitations** | **Inconsistency** | **Indirectness** | **Imprecision** | **Publication bias** | **Upward factors** | **Quality** |
| **3.1 Increased smoking increases the risk of female rheumatoid arthritis** | | | | | | | | | | | | | |
| Sugiyama 2010 | Smoking, Current and former smoking | 13 | 5 | 13885/  584455 | OR=1.27(1.12-1.44)  OR=1.31(1.12-1.54)  OR=1.22(1.06-1.40) | Significant | Serious limitations -1 | No serious inconsistency | No serious indirectness | No serious imprecision | Undetected | / | +  very low |
| Lahiri 2012 | Smoking | 4 | 4 |  |  | Significant | No serious limitations | No serious inconsistency | No serious indirectness | No serious imprecision | Serious publication bias -1 | / | +  very low |
| **3.2 Smoking has a lower impact on the prevalence of rheumatoid arthritis in women than in men** | | | | | | | | | | | | | |
| Sugiyama 2010 | Smoking, Current and former smoking | 12 | 5 | 13885/  584455 | OR=1.89(1.56-2.28)  OR=1.27(1.12-1.44) | Significant | Serious limitations -1 | No serious inconsistency | No serious indirectness | No serious imprecision | Undetected | / | +  very low |
| Lahiri 2012 | Smoking | 4 | 4 |  |  | Significant | No serious limitations | No serious inconsistency | No serious indirectness | No serious imprecision | Serious publication bias -1 | / | +  very low |

**Supplementary 1. Table 4 GRADE evidence profile: female chronic obstructive pulmonary disease**

| **Summary of findings Quality assessment** | | | | | | | | | | | | | |
| --- | --- | --- | --- | --- | --- | --- | --- | --- | --- | --- | --- | --- | --- |
| **Studies** | **Exposure** | **Case control** | **Cohort** | **Events/Total** | **Relative risk(95% CI)** | **Risk difference(95% CI)** | **Limitations** | **Inconsistency** | **Indirectness** | **Imprecision** | **Publication bias** | **Upward factors** | **Quality** |
| **4.1 Smoking increases the risk of female chronic obstructive pulmonary disease** | | | | | | | | | | | | | |
| Gan 2006 | Former and current smoking | 0 | 11 | ？/55709 |  |  | No serious limitations | Serious inconsistency -1 | Serious indirectness -1 | No serious imprecision | Undetected | Dose response+1  Mix response+1 | ++  Low |

**Supplementary 1. Table 5 GRADE evidence profile: female digestive system disease**

| **Summary of findings Quality assessment** | | | | | | | | | | | | | |
| --- | --- | --- | --- | --- | --- | --- | --- | --- | --- | --- | --- | --- | --- |
| **Studies** | **Exposure** | **Case control** | **Cohort** | **Events/Total** | **Relative risk(95% CI)** | **Risk difference(95% CI)** | **Limitations** | **Inconsistency** | **Indirectness** | **Imprecision** | **Publication bias** | **Upward factors** | **Quality** |
| **5.1 Smoking increases the risk of female pancreatitis** | | | | | | | | | | | | | |
| Andriulli 2010 | Smoking, Current and former smoking | 10 | 2 | 1705/  152746 | RR=2.1(1.2-6.9)  RR=2.8(1.4-5.6)  RR=1.3(0.6-1.3) | Significant | No serious limitations | No serious inconsistency | No serious indirectness | No serious imprecision | Undetected | Large effect+1 | +++  Moderate |
| Ye 2015 | Smoking, Current and former smoking | 14 | 6 | 4831/  330386 | RR=2.29(1.00-5.24)  RR=2.44(1.00-6.00)  RR=2.10(0.80-5.60) | Significant | No serious limitations | No serious inconsistency | No serious indirectness | No serious imprecision | Undetected | Large effect+1 | +++  Moderate |
| **5.2 Smoking increases the risk of female chronic pancreatitis** | | | | | | | | | | | | | |
| Ye 2015 | Smoking, Current and former smoking | 14 | 6 | 4831/  330386 | RR=1.29(1.76-3.64) | Significant | No serious limitations | No serious inconsistency | No serious indirectness | No serious imprecision | Undetected | Large effect+1 | +++  Moderate |
| **5.3 Smoking increases the risk of female pancreatic cancer** | | | | | | | | | | | | | |
| Lodice 2008 | Smoking | 47 | 35 | 10490/  537303 | RR=1.29(1.13-1.46) | Significant | No serious limitations | No serious inconsistency | No serious indirectness | No serious imprecision | Undetected | / | ++  Low |
| Lugo 2018 | Smoking, Current and former smoking | 38 | 40 | 45527/？ | RR=1.9(1.7-2.0)  RR=1.2(1.0-1.3)  RR=1.5(1.4-1.6) | Significant | No serious limitations | No serious inconsistency | No serious indirectness | No serious imprecision | Undetected | / | ++  Low |
| **5.4 Smoking increases the mortality of female pancreatic cancer** | | | | | | | | | | | | | |
| Ben 2019 | Current and former smoking | 0 | 20 | 15341/  2517623 | HR=1.82(1.68-1.98)  HR=1.15(1.05-1.26) | Significant | No serious limitations | Serious inconsistency -1 | No serious indirectness | No serious imprecision | Undetected | / | ++  Low |
| **5.5 Smoking increases the risk of female liver cancer** | | | | | | | | | | | | | |
| Lee 2009 | Smoking, Current and former smoking | 58 | 38 | 11359/  3873700 | RR=1.21(1.06-1.37)  RR=1.86(1.33-2.60)  RR=1.45(0.80-2.65) | Significant | No serious limitations | No serious inconsistency | No serious indirectness | No serious imprecision | Undetected | / | ++  Low |
| **5.6 Smoking increases the mortality of female liver cance** | | | | | | | | | | | | | |
| Pang 2015 | Current smoking | 0 | 27 | ？/3184616 | HR=1.85(1.2-1.84) | Significant | No serious limitations | No serious inconsistency | No serious indirectness | No serious imprecision | Undetected | / | ++  Low |
| **5.7 Smoking increases the risk of female gastric cancer** | | | | | | | | | | | | | |
| Ladeiras-Lopes 2008 | Current smoking | 5 | 27 | ? | RR=1.20(1.01-1.43) | Significant | No serious limitations | No serious inconsistency | No serious indirectness | No serious imprecision | Undetected | / | ++  Low |
| **5.8 Smoking increases the risk of female upper gastrointestinal cancer** | | | | | | | | | | | | | |
| Ansary-Moghaddam 2009 | Smoking | 15 | 72 | ？/3184616 | RR=2.18(2.13-3.7) | Significant | No serious limitations | No serious inconsistency | No serious indirectness | No serious imprecision | Undetected | Large effect+1 | +++  Moderate |
| **5.9 Smoking increases the risk of female colon cancer** | | | | | | | | | | | | | |
| Cheng 2015 | Current and former smoking | 0 | 24 | 14186/？ | RR=1.19(1.09-1.30)  RR=1.08(0.97-1.21) | Significant | No serious limitations | Serious inconsistency -1 | No serious indirectness | No serious imprecision | Undetected | / | ++  Low |
| Boterri 2008 | Smoking, Current and former smoking | 39 | 56 | 39 779/? | RR=1.14(1.03-1.25)  RR=1.05(0.94-1.18)  RR=1.20(1.11-1.30) | Significant | No serious limitations | No serious inconsistency | No serious indirectness | No serious imprecision | Undetected | / | ++  Low |
| Tsoi 2009 | Smoking | 0 | 29 | more than13000/  1463796 | RR=1.06(0.95-1.19) | No serious significant | No serious limitations | No serious inconsistency | No serious indirectness | Serious imprecision -1 | Undetected | / | +  Very low |
| **5.10 Smoking increases the risk of female rectal cancer** | | | | | | | | | | | | | |
| Cheng 2015 | Current and former smoking | 0 | 24 | 6814/？ | RR=1.27(1.05-1.52)  RR=1.16(0.97-1.40) | Significant | No serious limitations | Serious inconsistency -1 | No serious indirectness | No serious imprecision | Undetected | / | ++  Low |
| Boterri 2008 | Smoking, Current and former smoking | 39 | 56 | 39 779/? | RR=1.14(1.03-1.25)  RR=1.05(0.94-1.18)  RR=1.20(1.11-1.30) | Significant | No serious limitations | No serious inconsistency | No serious indirectness | No serious imprecision | Undetected | / | ++  Low |

| Tsoi 2009 | Smoking | 0 | 29 | more than13000/  1463796 | RR=1.06(0.95-1.19) | No serious significant | No serious limitations | No serious inconsistency | No serious indirectness | Serious imprecision -1 | Undetected | / | Very low + |
| --- | --- | --- | --- | --- | --- | --- | --- | --- | --- | --- | --- | --- | --- |
| **5.11 Smoking increases the risk of female diverticular disease** | | | | | | | | | | | | | |
| Aune 2017 | Smoking, Current and former smoking | 0 | 6 | 6076/  385291 | RR=1.38(1.15-1.66)  RR=1.28(1.06-1.54)  RR=1.31(1.16-1.49) | Significant | No serious limitations | No serious inconsistency | No serious indirectness | No serious imprecision | Undetected | / | ++  Low |
| **5.12 Smoking increases the risk of female colitis** | | | | | | | | | | | | | |
| Almomani 2020 | smoking | 3 | 1 | 1361/1725 | OR=2.93(2.09-4.1) | Significant | No serious limitations | Serious inconsistency -1 | No serious indirectness | No serious imprecision | Undetected | Dose effect+1 | ++  Low |

**Supplementary 1. Table 6 GRADE evidence profile: female mental disease**

| **Summary of findings Quality assessment** | | | | | | | | | | | | | |
| --- | --- | --- | --- | --- | --- | --- | --- | --- | --- | --- | --- | --- | --- |
| **Studies** | **Exposure** | **Case control** | **Cohort** | **Events/Total** | **Relative risk(95% CI)** | **Risk difference(95% CI)** | **Limitations** | **Inconsistency** | **Indirectness** | **Imprecision** | **Publication bias** | **Upward factors** | **Quality** |
| **6.1 Smoking increases the risk of female Alzheimer's disease** | | | | | | | | | | | | | |
| Zhong 2014 | Smoking, Current and former smoking | 0 | 22 | 5816/  960280 | RR=1.27(1.12-1.43)  RR=1.31(1.14-1.53)  RR=1.16(0.94-1.44) | Significant | Serious limitations -1 | No serious inconsistency | No serious indirectness | No serious imprecision | Undetected | / | +  Very low |
| **6.2 Smoking increases the risk of female all-cause dementia** | | | | | | | | | | | | | |
| Zhong 2014 | Smoking, Current and former smoking | 0 | 27 | 14935/  960280 | RR=1.21(1.03-1.43)  RR=1.30(1.11-1.52)  RR=1.10(0.92-1.32) | Significant | Serious limitations -1 | No serious inconsistency | No serious indirectness | No serious imprecision | Undetected | / | +  Very low |

**Supplementary 1. Table 7 GRADE evidence profile: non-pregnant female reproductive system disease**

| **Summary of findings Quality assessment** | | | | | | | | | | | | | |
| --- | --- | --- | --- | --- | --- | --- | --- | --- | --- | --- | --- | --- | --- |
| **Studies** | **Exposure** | **Case control** | **Cohort** | **Events/Total** | **Relative risk(95% CI)** | **Risk difference(95% CI)** | **Limitations** | **Inconsistency** | **Indirectness** | **Imprecision** | **Publication bias** | **Upward factors** | **Quality** |
| **7.1** **Smoking increases the risk of non-pregnant female endometrial cancer** | | | | | | | | | | | | | |
| Zhou 2008 | Ever smoking | 24 | 10 | / | RR=0.81(0.74-0.88)  RR=0.72(0.66-0.79) | Significant | No serious limitations | No serious inconsistency | No serious indirectness | No serious imprecision | Undetected | Mix effect+1 | +++  Moderate |
| **7.2 Smoking increases the risk of non-pregnant female cervical cancer** | | | | | | | | | | | | | |
| Han 2017 | Smoking | / | / | 128/323 | RR=3.88(2.13-7.08) | Significant | Serious limitations -1 | Serious inconsistency -1 | Serious indirectness -1 | No serious imprecision | Undetected | / | +  Very low |
| Sugawara 2019 | Smoking | 3 | 2 | 1316/  231001 | RR=2.03(1.49-2.57) | Significant | Serious limitations -1 | No serious inconsistency | No serious indirectness | No serious imprecision | Serious publication bias -1 | / | +  Very low |
| Kaderli 2014 | Smoking | 0 | 10 | ？/30924 | RR=1.5(1.4-1.7) | Significant | Serious limitations -1 | Serious inconsistency -1 | No serious indirectness | No serious imprecision | Undetected | / | +  Very low |

| **7.3** **Smoking increases the risk of non-pregnant female breast cancer** | | | | | | | | | | | | | |
| --- | --- | --- | --- | --- | --- | --- | --- | --- | --- | --- | --- | --- | --- |
| Macacu 2015 | Smoking, Current and former smoking | 44 | 31 | 665836/？ | RR=1.09(1.06-1.12) | Significant | No serious limitations | No serious inconsistency | No serious indirectness | No serious imprecision | Undetected | / | ++  Low |
| Sollie 2017 | Former and current smoking | 0 | 12 | 400944/？ | HR=1.02(0.93-1.12)  HR=1.28(1.17-1.41) | Significant | No serious limitations | Serious inconsistency -1 | No serious indirectness | No serious imprecision | Serious publication bias -1 | / | +  Very low |
| **7.4** **Smoking increases the risk of non-pregnant female serous or/and mucinous tumors of ovary** | | | | | | | | | | | | | |
| Santucci 2019 | Smoking, Current and former smoking, smoking density and smoking time | 31 | 6 | 70646/? | RR=1.78(1.52-2.07)  RR=1.05(0.64-1.17)  RR=1.07(0.98-1.18)  RR=1.12(1.00-1.25)  RR=1.34(1.16-1.55)  RR=1.06(1.00-1.13) | Significant | Serious limitations -1 | Serious inconsistency -1 | No serious indirectness | No serious imprecision | Undetected | Dose effect+1 | +  Very low |
| Jordan 2006 | Current and former smoking | 9 | 1 | 6921/  121360 | RR=1.0(0.9-1.2)  RR=1.0(0.8-1.2)  RR=2.1(1.7-2.7)  RR=1.1(0.9-1.4) | Significant | Serious limitations -1 | No serious inconsistency | No serious indirectness | No serious imprecision | Serious publication bias -1 | Dose effect+1 | +  Very low |
| Faber 2013 | Smoking, Current and former smoking, cigarettes consumption, the age of starting smoking and smoking time | 21 | 0 | 14724/  19066 | OR=1.31(1.03-1.65)  0R=0.92(0.76-1.10)  OR=0.99(0.83-1.17)  RR=1.05(0.97-1.14) | Significant | Serious limitations -1 | No serious inconsistency | No serious indirectness | No serious imprecision | Serious publication bias -1 | Dose effect+1 | +  Very low |

**Supplementary 1. Table 8 GRADE evidence profile: pregnant women and their fetuses**

| **Summary of findings** | | | | | | | **Quality assessment** | | | | | | |
| --- | --- | --- | --- | --- | --- | --- | --- | --- | --- | --- | --- | --- | --- |
| **Studies** | **Exposure** | **Case control** | **Cohort** | **Events/Total** | **Relative risk(95% CI)** | **Risk difference(95% CI)** | **Limitations** | **Inconsistency** | **Indirectness** | **Imprecision** | **Publication bias** | **Upward factors** | **Quality** |
| **8.1 Smoking can adversely affect the various stages of pregnancy in female reproduction (folliculogenesis, steroidogenesis, preimplantation embryo development, Fallopian tubes, embryo implantation, uterine flow velocity and myometrial activity)** | | | | | | | | | | | | | |
| Dechanet 2011 | smoking | ? | ? | ? | ? | ? | Serious limitations -1 | Serious inconsistency -1 | No serious indirectness | Serious imprecision -1 | Serious publication bias -1 | / | +  Very low |
| **8.2 Smoking increases the chance of spontaneous abortion in pregnant women** | | | | | | | | | | | | | |
| Hughes 1996 | Smoking | 6 | 1 | ?/97762 | ? | ? | Serious limitations -1 | Serious inconsistency -1 | No serious indirectness | Serious imprecision -1 | Serious publication bias -1 | / | +  Very low |
| **8.3 Smoking increases the risk of postpartum depression in pregnant women** | | | | | | | | | | | | | |
| Chen 2019 | smoking | 4 | 4 | ?/1476922 | OR=2.31(1.92-2.81) | Significant | No serious limitations | Serious inconsistency -1 | No serious indirectness | No serious imprecision | Undetected | Large effect+1 | ++  Low |

| **8.4 Smoking increases the risk of fetal schizophrenia in pregnant women** | | | | | | | | | | | | | |
| --- | --- | --- | --- | --- | --- | --- | --- | --- | --- | --- | --- | --- | --- |
| Hunter 2018 | Smoking | 3 | 4 | 10278/  2444910 | RR=1.29(1.1-1.51) | Significant | No serious limitations | Serious inconsistency -1 | No serious indirectness | No serious imprecision | Undetected | / | +  Very low |
| **8.5 Smoking increases the risk of fetal attention deficit and hyperactivity disorder in pregnant women** | | | | | | | | | | | | | |
| Dong 2017 | Smoking during pregnancy | ? | ? | ?/3076173 | OR=1.775(1.324-2.379) | Significant | No serious limitations | Serious inconsistency -1 | No serious indirectness | No serious imprecision | Undetected | / | +  Very low |
| Huang 2018 | Smoking during pregnancy | 5 | 15 | 50044/  2998059 | OR=1.60(1.45-1.76) | Significant | No serious limitations | No serious inconsistency | No serious indirectness | No serious imprecision | Undetected | Dose effect+1 | +++  Moderate |
| **8.6 Smoking increases the risk of fetal coronary heart disease in pregnant women** | | | | | | | | | | | | | |
| Lee 2013 | Smoking during pregnancy | 23 | 5 | ? | RR=1.11(1.02-1.21) | Significant | Serious limitations -1 | Serious inconsistency -1 | No serious indirectness | No serious imprecision | Undetected | / | +  Very low |
| Zhang 2017 | Smoking during pregnancy | 38 | 5 | 74366/？ | RR=1.11(1.04-1.18) | Significant | No serious limitations | Serious inconsistency -1 | No serious indirectness | No serious imprecision | Serious publication bias -1 | Dose effect+1 | +  Very low |

## Evidence summary of core information

## Section 1 The correlation between smoking and risks of female cardiovascular disease

Smoking and passive smoking are recognized as independent risk factors for such diseases1. The global mortality of cardiovascular diseases has increased by 12.5% over the past decade, and cardiovascular diseases currently account for about one-third of global deaths^2^, so preventing cardiovascular diseases will greatly improve global health. Smoking can cause serious damage to the body and even affect all stages of arteriosclerosis by increasing the oxidative stimulation including inflammation, thrombosis and oxidation of low-density lipoprotein cholesterol^[55]^

There are 2 articles examining the effect of smoking on coronary heart disease. Woodward3 found that smoking increases the mortality of female coronary heart disease (HR=1.73, 95%CI: 1.44-1.70). Hackshaw^4^ studied the dose-response relationship between the amount of smoking and the risk of coronary heart disease and the results showed that the women who smoked 1 cigarette per day had a 57% increased risk of coronary heart disease (RR=1.57, 95%CI: 1.29-1.91), Smoking 5 cigarettes is associated with a 76% increased risk (RR=, 95%CI: 1.46-2.13), and smoking 20 cigarettes per day has a 2.84 times higher risk in women (RR=2.84, 95%CI: 2.21-3.64).

Huxley^5^ studied the relationship between smoking and mortality from heart failure and showed that smoking increased the risk of death from heart failure in women with good significance (HR=1.39, 95% CI: 1.02-1.89)

Aune^6^ studied the relationship between smoking and the risk of developing sudden cardiac death, which was 2.44 times greater for current smokers than for the normal female population (95% CI: 1.80-3.31) and increased by 40% for former smokers (95% CI: 1.10-1.79).

Three articles examined the effect of smoking on stroke in women. Woodward3 examined the relationship between smoking and the risk of death from stroke in women, which showed that smoking increased the risk of death from stroke by 42% (HR=1.42, 95% CI: 1.26-1.62). Peters^7^ studied the relationship between smoking and the risk of stroke in women, which showed that smoking was a risk factor for stroke (RR=1.83, 95% CI: 1.22-1.70). Hackshaw^4^ demonstrated a dose-response relationship between smoking and the risk of stroke in women, with a 31% (95% CI: 1.13-1.52) increased risk for 1 cigarette per day and a 44% (95% CI: 1.30-2.34) increased risk for 5 cigarettes per day, and for 20 cigarettes per day, the risk was 2.16 times higher for women (95% CI: 1.69-2.75), and for 20 cigarettes per day, the risk was 2.16 times higher for women (95% CI: 1.69-2.75).

Hackshaw^4^ researched the dose-response relationship between smoking and the overall risk of cardiovascular events and found that women had a 65% (95% CI: 1.13-2.40) increased risk for 1 cigarette per day, a 74% (95% CI: 1.30-2.34) increased risk for 5 cigarettes per day, and a 2.16 times (95% CI: 1.69-2.76) increased risk for 20 cigarettes per day compared to the general female population.

## Section 2 The correlation between smoking and risks of female diabetes

The prevalence of type 2 diabetes (T2D) has gradually increased over the past three decades and has become a major global public health challenge. Given the high prevalence of smoking in many countries and the increasing burden of diabetes worldwide, reducing tobacco use should be prioritised as an important public health strategy that may contribute to the prevention and control of diabetes^8^. Two studies have shown that smoking increases the risk of diabetes in women, with results of 1.42 (95%: 1.19, 1.69) and 1.33 (95% : 1.26-1.41) relative risks of diabetes for current smokers respectively^8^, ^9^.

## Section 3 The correlation between smoking and risks of female rheumatoid arthritis

Rheumatoid arthritis (RA) is a major autoimmune disease and is typically characterised by chronic inflammation of the articulations and bone destruction^10^. As the most common form of inflammatory arthritis, its worldwide prevalence is approximately 1%, with women at two to three times the risk of developing rheumatoid arthritis compared to men due to the effect of oestrogen on the immune system. The cumulative adult prevalence is 3.6% for women and 1.7% for men^11^.

Two systematic reviews have examined the effect of smoking on morbidity and mortality in women with rheumatoid arthritis, and both studies suggest that smoking is an important environmental risk factor for developing rheumatoid arthritis^12, 13^.

A study of Sugiyama et al. (2010) stratified by sex, smoking status (never, ever, and current), and annual cigarette consumption showed that the risk of rheumatoid arthritis was approximately two times greater for male smokers than for nonsmokers, and about 1.3 times greater for female smokers, that is, for men, the overall ORs for rheumatoid arthritis among never, current, and ever smokers were 1.89 (95% CI, 1.56-2.28), 1.87 (95% CI, 1.49-2.34) and 1.76 (95% CI, 1.33-2.31), while the ORs for rheumatoid arthritis in women were 1.27 (95% CI, 1.12-1.44), 1.31 (95% CI, 1.12-1.54), 1.22 (95％CI, 1.06-1.40) respectively, slightly lower than for men45. In 2012, a meta-analysis of 11 case-control studies and 5 cohort studies by Lahiri et al. showed that smoking was a risk factor for rheumatoid arthritis and it was dose-related, with male smokers at greater risk than female smokers (OR=3.91, 95% CI 2.78-5.50)^13^. This difference between the sexes may be due to three factors: (1) hormonal effects^14^; (2) occupational factors, for example, Stolt et al^15^ reported that silica exposure was associated with an increased risk of rheumatoid arthritis in men and men were more likely to be exposed to silica compared to women due to occupational factors; (3) differences in smoking intensity between men and women, with men smoking intensity is greater in men than in women^13^.

## Section 4 The correlation between smoking and risks of female respiratory disease

Smoking was the leading risk factor for chronic respiratory diseases, For women, the leading cause of smoking-attributable DALYs was COPD1. Gan found that female current smokers had with increasing age a significantly faster annual decline in FEV1% predicted than male current smokers (linear regression analysis, R2 = 0.56; p = 0.008)^16^.

## Section 5 The correlation between smoking and risks of female digestive system disease

A total of 14 articles reported the effect of smoking on morbidity and mortality from digestive diseases, with a moderate risk of bias. Two of these articles reported the effect of smoking on the risk of pancreatitis in women and the results were highly consistent: the study of Andriulli^17^ indicated that female ever smokers and current smokers substantially increased the risk of pancreatitis, with RR=2.1, 95% CI: 1.2-6.9 and RR=2.8, 95% CI: 1.4-5.6, respectively. However, the increased risk of pancreatitis in former smokers was not statistically significant (RR=1.3, 95% CI:0.6-1.3), and similar results were obtained in the article from Ye^18^, with RR=2.29, 95% CI:1.00-5.24 for pancreatitis in ever smokers and RR=2.44, 95% CI:1.00-6.00 in current smokers. The increased risk of pancreatitis in former smokers was not statistically significant (RR=2.10, 95% CI: 0.80-5.60), suggesting that smoking is a significant risk factor for the development of pancreatitis in women, and also that quitting smoking significantly reduces the risk. A study by Ye1^8^ has reported results across pancreatitis subtypes, and for acute pancreatitis, there was no statistically significant effect of smoking (ever smokers: RR= 1.11, 95% CI: 0.82-1.52; current smokers: RR=1.25, 95% CI: 0.97-2.13; former smokers: RR=1.15, 95% CI: 0.79-1.67), but for chronic pancreatitis, smoking was able to significantly increase the risk of its development (ever smokers: RR=1.79, 95 CI:1.22- 2.64; current smokers: RR=2.53, 95% CI: 1.76-3.64; former smokers: RR=1.26, 95% CI: 0.84-1.88)

Two articles reported the effect of smoking on the risk of pancreatic cancer in females. the article by Iodice^19^ demonstrated that smoking can increase the risk of pancreatic cancer in females (RR=1.29, 95% CI: 1.13-1.46). Lugo^20^ reported the results based on the classification of smoking status among current smokers (RR=1.9, 95% CI:1.7-2.0), former smokers (RR=1.2, 95% CI:1.0-1.3), and ever smokers (RR=1.5, 95% CI:1.4-1.6). In a study by Ben^21^, smoking also increased the risk of death from pancreatic cancer in women, with a 15% increased risk of death from pancreatic cancer in former smokers (HR=1.15, 95% CI:1.05-1.26) and an 82% increased risk of death in current smokers (HR=1.82, 95% CI:1.68-1.98), which was good for significance.

One article examined the relationship between smoking and the risk of liver cancer in women. The results of a study by Lee^22^ showed a significant increase in the prevalence of liver cancer in ever smokers and current smokers, with effect values of RR=1.21, 95% CI: 1.06-1.37 and RR=1.86, 95% CI: 1.33-2.60, respectively, however for former smokers this effect was not statistically (RR=1.45, 95% CI: 0.80-2.65). A study by Pang^23^ showed that smoking substantially increased the risk of death from liver cancer (HR=1.85, 95% ci: 1.20-1.84).

Two articles reported about the impact of smoking on the risk of colorectal cancer in women, however the findings of these two articles differed. Botteri's^24^ study concluded that for current smokers the effect of smoking on colorectal cancer was not statistically significant (RR=1.05, 95% CI: 0.94-1.18), whereas for ever smokers and former smokers smoking was able to increase the risk. The study by Tsoi^25^ showed that the current evidence did not prove that smoking was a risk factor for colorectal cancer in women (RR=1.06, 95% CI: 0.95-1.19). Cheng^26^ studied the effect of smoking on the risk of colon and rectal cancer in women and showed that the effect on the risk of colon and rectal cancer in current smokers was not statistically significant (RR=1.08, 95% CI: 0.97-1.21 and RR=1.16, 95% CI: 0.97-1.40, respectively), whereas former smokers increased the risk of colon and rectal cancer with good significance(RR=1.19, 95% CI: 1.09-1.30 and RR=1.27, 95% CI: 1.05-1.52 respectively). The findings of the above studies are consistent in suggesting that ever smoking has a greater effect on colorectal cancer than current smoking, however it is generally accepted that current smokers smoke to a greater extent than former smokers, therefore further quantitative studies are needed to investigate the dose-response relationship between the amount of smoking and the risk of colorectal cancer.

Aune^27^ showed that smoking increased the risk of diverticular disease in women with good significance (current smokers: RR=1.38, 95% CI: 1.15-1.66, former smokers: RR=1.28, 95% CI: 1.06-1.54, ever smokers: RR=1.31, 95% CI: 1.16-1.49), and A study by Ladeiras-Lopes^28^ showed an increased risk of gastric cancer in women current smokers with good significance (RR=1.20, 95% CI: 1.01-1.43) and Ansary-Moghaddam2^9^ found that smoking can substantially increase the risk of upper gastrointestinal cancer with good significance (RR=2.81, 95% CI: 2.13-3.70), Al Momani3^0^ found that smoking substantially increased the risk of microscopic colitis with good significance (OR=2.93, 95% CI: 2.09-4.10).

## Section 6 The correlation between smoking and risks of female mental disease

Mental illness have a huge burden on health in the world today. According to statistics, the global burden of mental illness accounts for 32·4% of years lived with disability (YLDs) and 13·0% of disability-adjusted life-years (DALYs)^31^. Smoking rate was high in patients with mental illness, and it was estimated that more than 200,000 of 520,000 people suffer from mental illness who died mainly from chronic diseases caused by smoking in the United States^32^. Smoking groups were more likely to be discriminated against and stigmatized in social life, causing further damage to their mental health^33^. At the same time, smoking also directly increased the risk of certain mental and psychiatric diseases, which promoted the pathological process of Alzheimer’s disease (AD) through brain oxidative stress, thereby increasing the risk of AD^34^. It was indicated that fomer female smokers suffered from all-cause dementia (RR=1.21, 95%CI: 1.03-1.43) and AD (RR=1.27, 95%CI: 1.12-1.43, I2=0 %), and female current smokers also showed increased risks of all-cause dementia (RR=1.30, 95%CI: 1.11-1.52) and AD (RR=1.32, 95%CI: 1.14-1.53)^35^.However, only one literature was included in this systematic review and a heterogeneity analysis could not be conducted, which result in a greater risk of bias.

## Section 7 The correlation between smoking and risks of non-pregnant female reproductive system disease

Female reproductive system diseases mainly include gynaecological inflammation, gynaecological tumours, menstrual disorders and infertility, which bring both physical and mental effects to women and seriously reduce their quality of life. According to WHO global report on trends in prevalence of tobacco use 2000-2025, there were as many as 244 million women using tobacco in 2018^36^. It has been shown that cigarette smoke contains a variety of toxins that affect reproductive function to varying degrees^3^7, and smoking is therefore considered to be an important factor in the increased prevalence and mortality of female reproductive disorders.

Seven systematic reviews have examined the effects of smoking on the prevalence and mortality of reproductive disorders in non-pregnant women^38-45^.

Two systematic reviews directly showed that smoking increased the risk of cervical cancer^38, 39^, with an RR=2.03 (95%:1.49-2.57) and OR=1.5 (95%CI:1.4-1.7), respectively. One study indirectly showed that smoking increased the risk of cervical cancer. A meta-analysis of 1 cross-sectional study and 19 case-control studies by Han et al. in 2017 found that P16INK4a hypermethylation was significantly associated with an increased risk of cervical cancer (CC) (OR = 12.17, 95% CI:5.86-25.27) and there was also a significant association between P16INK4a hypermethylation and smoking habits (OR = 3.88, 95% CI:2.13-7.08), thus demonstrating that smoking increases the risk of cervical cancer^40^.

Two studies validated the association between smoking and breast cancer. A meta-analysis of 27 prospective studies found an SRR of 1.10 (95% CI:1.09-1.12) for breast cancer for regular smokers, indicating a modest increase in the risk of breast cancer in women who smoke4^1^. Sollie et al.found that hazard ratio (HR) for breast cancer associated death in former smokers was 1.02 (0.93, 1.12) and for current smokers 1.28 (1.17, 1.41) when compared to never smokers^42^.

Three studies showed that smoking increases the risk of non-pregnant female serous or/and mucinous tumors of ovary. Jordan^43^ reported that there was a significant doubling of risk of mucinous ovarian cancer in current smokers compared to never smokers (summary RR 2.1, 95%CI 1.7–2.7) as well as a dose-response relationship between mucinous cancer and smoking, but no increased risk of serous (1.0, 95%CI 0.8–1.2). Meanwhile, Santucci’ study^44^ confirmed this conclusion that the summary RRs were 1.78 (95% CI 1.52–2.07) for mucinous and 1.05 (95% CI 0.94; 1.17) for serous cancer. And there is a strong dose-risk relationship with mucinous ovarian cancer whose risk was noticeably higher with smoking intensity and duration (RR: 2.35 for 20 cigarettes/day, and 2.11 for 20 years of smoking). Faber^45^ found that current cigarette smoking increased the risk of invasive mucinous (OR = 1.31; 95 % CI: 1.03–1.65) and borderline mucinous ovarian tumors (OR = 1.83; 95 % CI: 1.39–2.41), while former smoking increased the risk of borderline serous ovarian tumors (OR = 1.30; 95 % CI: 1.12–1.50).

## Section 8 Effects of smoking on pregnant women and their fetuses

Considering that pregnancy is a special period for women, pregnant women and fetuses are more susceptible to external influences during this period. Studies shown that active smoking or passive smoking of women during pregnancy was harmful to mothers and fetuses^46^-^52^. Smoking during pregnancy might increase the risk of postpartum depression in women (OR=2.32, 95%CI: 1.92-2.81, I2=77.2%)^46^, fetal schizophrenia (RR=1.29, 95%CI: 1.10-1.51, I2=71%)^47^ and fetal attention deficit and hyperactivity disorder (OR=1.775, 95% CI: 1.324-2.379)^48^. Lee^49^ pointed out that smoking caused coronary heart disease in pregnant women and brought about congenital heart disease in their offspring (RR=1.11, 95% CI: 1.02-1.21). Dechanet^50^ reported that smoking could adversely affect the various stages of pregnancy in female reproduction including folliculogenesis, steroidogenesis, preimplantation embryo development, fallopian tubes, embryo implantation, uterine flow velocity and myometrial activity. Hughes^51^ found that smoking might increase the chance of spotaneous abortion in pregnant women. But both Dechanet and Hughes did not do data merging and meta-analysis, which resulted in low quality articles. In addition, passive smokers might also have similar adverse effects5^2^.

## reference

1. GBD 2015 Tobacco Collaborators. Smoking prevalence and attributable disease burden in 195 countries and territories, 1990-2015: a systematic analysis from the Global Burden of Disease Study 2015 [published correction appears in Lancet. 2017 Oct 7;390(10103):1644]. Lancet. 2017;389(10082):1885-1906. doi:10.1016/S0140-6736(17)30819-X

Joseph P, Leong D, McKee M, et al. Reducing the Global Burden of Cardiovascular Disease, Part 1: The Epidemiology and Risk Factors. Circ Res. 2017;121(6):677-694. doi:10.1161/CIRCRESAHA.117.308903

Woodward M, Lam TH, Barzi F, et al., Smoking, quitting, and the risk of cardiovascular disease among women and men in the Asia-Pacific region. Int J Epidemiol. 2005;34(5): 1036-45.

Hackshaw A, Morris JK, Boniface S, Tang JL, Milenković D. Low cigarette consumption and risk of coronary heart disease and stroke: meta-analysis of 141 cohort studies in 55 study reports [published correction appears in BMJ. 2018 Apr 11;361:k1611] [published correction appears in BMJ. 2018 Nov 28;363:k5035]. BMJ. 2018;360:j5855. Published 2018 Jan 24. doi:10.1136/bmj.j5855

Huxley RR, Woodward M. Cigarette smoking as a risk factor for coronary heart disease in women compared with men: a systematic review and meta-analysis of prospective cohort studies. Lancet. 2011;378:1297–305

Aune D, Schlesinger S, Norat T, et al. Tobacco smoking and the risk of sudden cardiac death: a systematic review and meta-analysis of prospective studies. Eur J Epidemiol. 2018;33(6):509-521.

Peters SA, Huxley RR, Woodward M. Woodward. Smoking as a risk factor for stroke in women compared with men: a systematic review and meta-analysis of 81 cohorts, including 3,980,359 individuals and 42,401 strokes. Stroke. 2013;44(10):2821-8.

Pan A, Wang Y, Talaei M, Hu FB, Wu T. Relation of active, passive, and quitting smoking with incident type 2 diabetes: a systematic review and meta-analysis. Lancet Diabetes Endocrinol. 2015;3(12):958-967. doi:10.1016/S2213-8587(15)00316-2

Akter S, Goto A, Mizoue T. Smoking and the risk of type 2 diabetes in Japan: A systematic review and meta-analysis. J Epidemiol. 2017;27(12):553-561. doi:10.1016/j.je.2016.12.017

Scott DL, Wolfe F, Huizinga TW. Rheumatoid arthritis. Lancet. 2010;376(9746):1094-1108. doi:10.1016/S0140-6736(10)60826-4

Smolen JS, Aletaha D, Barton A, et al. Rheumatoid arthritis. Nat Rev Dis Primers. 2018;4:18001. Published 2018 Feb 8. doi:10.1038/nrdp.2018.1

Sugiyama D, Nishimura K, Tamaki K, et al. Impact of smoking as a risk factor for developing rheumatoid arthritis: a meta-analysis of observational studies. Ann Rheum Dis. 2010;69(1):70-81. doi:10.1136/ard.2008.096487

Lahiri M, Morgan C, Symmons DP, Bruce IN. Modifiable risk factors for RA: prevention, better than cure?. Rheumatology (Oxford). 2012;51(3):499-512. doi:10.1093/rheumatology/ker299

Rayman M, Callaghan A. Nutrition and arthritis. Oxford, UK: Blackwell Publishing, 2006.

Stolt P, Kallberg H, Lundberg I, et al, EIRA study group. Silica exposure is associated with increased risk of developing rheumatoid arthritis: results from the Swedish EIRA study. Ann Rheum Dis. 2005;62:582–6.

Gan WQ, Man SF, Postma DS, Camp P, Sin DD. Female smokers beyond the perimenopausal period are at increased risk of chronic obstructive pulmonary disease: a systematic review and meta-analysis. Respir Res. 2006;7(1):52. Published 2006 Mar 29. doi:10.1186/1465-9921-7-52

Andriulli A, Botteri E, Almasio PL, et al. Smoking as a cofactor for causation of chronic pancreatitis: a meta-analysis. Pancreas. 2010;39(8):1205-1210.

Ye X, Lu G, Huai J, Ding J. Impact of smoking on the risk of pancreatitis: a systematic review and meta-analysis. PLoS One. 2015;10(4):e0124075. Published 2015 Apr 16. doi:10.1371/journal.pone.0124075

Iodice S, Gandini S, Maisonneuve P, et al. Tobacco and the risk of pancreatic cancer: a review and meta-analysis. Langenbecks Arch Surg. 2008;393(4):535-545.

Lugo A, Peveri G, Bosetti C, et al. Strong excess risk of pancreatic cancer for low frequency and duration of cigarette smoking: A comprehensive review and meta-analysis. Eur J Cancer. 2018;104:117-126.

Ben QW, Liu J, Sun YW, Wang LF, Zou DW, Yuan YZ. Cigarette Smoking and Mortality in Patients With Pancreatic Cancer: A Systematic Review and Meta-analysis. Pancreas. 2019;48(8):985-995.

Lee YC, Cohet C, Yang YC, et al. Meta-analysis of epidemiologic studies on cigarette smoking and liver cancer. Int J Epidemiol. 2009;38(6):1497-1511.

Pang Q, Qu K, Zhang J, et al. Cigarette smoking increases the risk of mortality from liver cancer: A clinical-based cohort and meta-analysis. J Gastroenterol Hepatol. 2015;30(10):1450-1460.

Botteri E, Iodice S, Bagnardi V, et al. Smoking and colorectal cancer: a meta-analysis. JAMA. 2008;300(23):2765-78.

Tsoi KK, Pau CY, Wu WK, Chan FK, Griffiths S, Sung JJ. Cigarette smoking and the risk of colorectal cancer: a meta-analysis of prospective cohort studies. Clin Gastroenterol Hepatol. 2009;7(6):682-688.e6885.

Cheng J, Chen Y, Wang X, et al. Meta-analysis of prospective cohort studies of cigarette smoking and the incidence of colon and rectal cancers[J]. Eur J Cancer Prev. 2015;24(1):6-15.

Aune D, Sen A, Leitzmann MF, Tonstad S, Norat T, Vatten LJ. Tobacco smoking and the risk of diverticular disease - a systematic review and meta-analysis of prospective studies. Colorectal Dis. 2017;19(7):621-633.

Ladeiras-Lopes R, Pereira AK, Nogueira A, et al. Smoking and gastric cancer: systematic review and meta-analysis of cohort studies. Cancer Causes Control. 2008;19(7):689-701.

Ansary-Moghaddam A, Huxley RR, Lam TH, Woodward M. The risk of upper aero digestive tract cancer associated with smoking, with and without concurrent alcohol consumption. Mt Sinai J Med. 2009;76(4):392-403.

Al Momani L, Balagoni H, Alomari M, et al. The association between smoking and both types of microscopic colitis: A systematic review and meta-analysis. Arab J Gastroenterol. 2020;21(1):9-18. doi:10.1016/j.ajg.2020.01.004

Vigo D, Thornicroft G, Atun R. Estimating the true global burden of mental illness. Lancet Psychiatry. 2016;3(2):171-178. doi:10.1016/S2215-0366(15)00505-2

Colton CW, Manderscheid RW. Congruencies in increased mortality rates, years of potential life lost, and causes of death among public mental health clients in eight states. Prev Chronic Dis. 2006;3(2):A42.

Brown-Johnson CG, Cataldo JK, Orozco N, Lisha NE, Hickman NJ 3rd, Prochaska JJ. Validity and reliability of the Internalized Stigma of Smoking Inventory: An exploration of shame, isolation, and discrimination in smokers with mental health diagnoses. Am J Addict. 2015;24(5):410-418. doi:10.1111/ajad.12215

Durazzo TC, Mattsson N, Weiner MW; Alzheimer's Disease Neuroimaging Initiative. Smoking and increased Alzheimer's disease risk: a review of potential mechanisms. Alzheimers Dement. 2014;10(3 Suppl):S122-S145. doi:10.1016/j.jalz.2014.04.009

Zhong G, Wang Y, Zhang Y, et al. Smoking is associated with an increased risk of dementia: a meta-analysis of prospective cohort studies with investigation of potential effect modifiers [published correction appears in PLoS One, 2015, 10(4):e0126169]. PLoS One. 2015;10(3):e0118333.

WHO global report on trends in prevalence of tobacco use 2000-2025, fourth edition. Geneva: World Health Organization; 2021. Licence: CC BY-NC-SA 3.0 IGO.

Hughes EG, Brennan BG. Does cigarette smoking impair natural or assisted fecundity?. Fertil Steril. 1996;66(5):679-689. doi:10.1016/s0015-0282(16)58618-x

Sugawara Y, Tsuji I, Mizoue T, et al. Cigarette smoking and cervical cancer risk: an evaluation based on a systematic review and meta-analysis among Japanese women. Jpn J Clin Oncol. 2019;49(1):77-86.

Kaderli R, Schnüriger B, Brügger LE. The impact of smoking on HPV infection and the development of anogenital warts. Int J Colorectal Dis. 2014;29(8):899-908.

Han YD, Wang XB, Cui NH, Zhang S, Wang C, Zheng F. Associations of P16INK4a promoter hypermethylation with squamous intra-epithelial lesion, cervical cancer and their clinicopathological features: a meta-analysis. Oncotarget. 2017;8(1):1871-1883.

Macacu A, Autier P, Boniol M, Boyle P. Active and passive smoking and risk of breast cancer: a meta-analysis. Breast Cancer Res Treat. 2015;154(2):213-224.

Sollie M, Bille C. Smoking and mortality in women diagnosed with breast cancer-a systematic review with meta-analysis based on 400,944 breast cancer cases. Gland Surg. 2017;6(4):385-393. doi:10.21037/gs.2017.04.06

Jordan SJ, Whiteman DC, Purdie DM, Green AC, Webb PM. Does smoking increase risk of ovarian cancer? A systematic review. Gynecol Oncol. 2006;103(3):1122-1129. doi:10.1016/j.ygyno.2006.08.012

Santucci C, Bosetti C, Peveri G, et al. Dose-risk relationships between cigarette smoking and ovarian cancer histotypes: a comprehensive meta-analysis. Cancer Causes Control. 2019;30(9):1023-1032. doi:10.1007/s10552-019-01198-8

Faber MT, Kjær SK, Dehlendorff C, et al. Cigarette smoking and risk of ovarian cancer: a pooled analysis of 21 case-control studies. Cancer Causes Control. 2013;24(5):989-1004. doi:10.1007/s10552-013-0174-4

Chen HL, Cai JY, Zha ML, Shen WQ. Prenatal smoking and postpartum depression: a meta-analysis. J Psychosom Obstet Gynaecol. 2019;40(2):97-105. doi:10.1080/0167482X.2017.1415881

Hunter A, Murray R, Asher L, Leonardi-Bee J. The Effects of Tobacco Smoking, and Prenatal Tobacco Smoke Exposure, on Risk of Schizophrenia: A Systematic Review and Meta-Analysis. Nicotine Tob Res. 2020;22(1):3-10. doi:10.1093/ntr/nty160

Dong T, Hu W, Zhou X, et al. Prenatal exposure to maternal smoking during pregnancy and attention-deficit/hyperactivity disorder in offspring: A meta-analysis. Reprod Toxicol. 2018;76:63-70. doi:10.1016/j.reprotox.2017.12.010

Lee LJ, Lupo PJ. Maternal smoking during pregnancy and the risk of congenital heart defects in offspring: a systematic review and metaanalysis. Pediatr Cardiol. 2013;34(2):398-407. doi:10.1007/s00246-012-0470-x

Dechanet C, Anahory T, Mathieu Daude JC, et al. Effects of cigarette smoking on reproduction. Hum Reprod Update. 2011;17(1):76-95. doi:10.1093/humupd/dmq033

Hughes EG, Brennan BG. Does cigarette smoking impair natural or assisted fecundity?. Fertil Steril. 1996;66(5):679-689. doi:10.1016/s0015-0282(16)58618-x

Shulman A, Ellenbogen A, Maymon R, Bahary C. Smoking out the oestrogens. Hum Reprod. 1990;5(3):231-233. doi:10.1093/oxfordjournals.humrep.a137077

The questionnaire design includes general information and Evidence-Based Core Information Demand Questionnaire.

The general information questionnaire included: age, gender, place of residence, educational level, marital status, occupation (health care workers, non-health care workers), smoking and chronic diseases.

The Evidence-Based Core Information Demand Questionnaire included cardiovascular diseases, digestive system diseases, rheumatoid arthritis, non-pregnant female reproductive system diseases, pregnant women and fetuses, and other 43 items in 6 dimensions. A 5-point scale was used to assess the degree of public demand (1=none, 2=low, 3=moderate, 4=high, 5=very high), with higher scores indicating higher levels of public needs . The overall Cronbach'α of the questionnaire was 0.995, and the Cronbach'α coefficients of each part were: cardiovascular 0.982, rheumatoid 0.980, digestive system 0.989, non-pregnant women reproductive system diseases 0.989, pregnant women and fetuses 0.979, and others 0.943. The content validity of the scale was assessed by Delphi expert consultation, and the Kendall harmony coefficient was 0.272 (P<0.01) in the first round of consultation and 0.101 (P<0.01) in the second round. The questionnaire has good reliability and validity.

A total of 681 questionnaires were distributed in this study to verify the completeness of the questionnaire content. The complete answer sheet and the time for answering the questionnaire > 2 minutes were considered as valid questionnaires. Finally, 670 questionnaires were included in the study, of which 9 questionnaires did not agree to participate in the survey, and 2 questionnaires were invalid questionnaires. The effective rate of the questionnaire was 98.3%.

Among the 670 members of the public who participated in the survey, 68.1% lived in cities, and 31.9% lived in rural areas; 397 were women (59.3%), and 273 were men (40.7%); the majority were younger than 26 years old, accounting for 42.1% ; The majority of those with education level are college/undergraduate or above (64.9%), followed by junior high school/secondary school/high school (31.3%); in terms of marital status, 53.6% are married and 44.9% are unmarried. In terms of occupation, non-medical workers are the main ones (87.8%). Non-smokers and those without chronic diseases accounted for 80.0% of those who smoked and those who had chronic diseases. The results of univariate analysis on the scores of the unmet needs of the female-related tobacco control information among the public with different characteristics: the public's gender, age, education level, occupation, smoking status, and chronic diseases have differences in the total score of the unmet needs for information. Statistical significance (P<0.05).

**Supplementary 2. Table 1 Univariate analysis of demand for core information**

| **Item** | **N（%）** | **Score** | **t/F** | **P** |
| --- | --- | --- | --- | --- |
| **Area** |  |  | -0.009 | 0.993 |
| Urban | 456（68.1） | 133.40±48.58 |  |  |
| Rural | 214（31.9） | 133.43±52.00 |  |  |
| **Sex** |  |  | -4.677 | **<0.001** |
| Male | 273（40.7） | 122.75±51.37 |  |  |
| Female | 397（59.3） | 140.74±47.13 |  |  |
| **Age** |  |  | 4.085 | **0.017** |
| <26 | 282（42.1） | 130.49±50.62 |  |  |
| 26-45 | 224（33.4） | 141.00±47.68 |  |  |
| >45 | 164（24.5） | 128.07±49.71 |  |  |
| **Education** |  |  | 5.543 | **0.004** |
| Elementary school and below | 25 （3.7） | 106.04±40.43 |  |  |
| Junior High School/Secondary School/High School | 210 (31.3） | 129.56±50.69 |  |  |
| College/Undergraduate and above | 435（64.9） | 136.84±49.10 |  |  |
| **Marital status** |  |  | 1.265 | 0.283 |
| Unmarried | 301（44.9） | 130.36±50.79 |  |  |
| Married | 359（53.6） | 135.60±49.01 |  |  |
| Others | 10（1.5） | 146.50±32.88 |  |  |
| **Profession** |  |  | 2.208 | **0.028** |
| Medical worker | 82 （12.2） | 144.72±49.31 |  |  |
| Non-medical worker | 588（87.8） | 131.83±49.54 |  |  |
| **Smoking** |  |  | -5.196 | **<0.001** |
| Yes | 134（20.0） | 113.85±46.22 |  |  |
| No | 536（80.0） | 138.30±49.32 |  |  |
| **Chronic disease** |  |  | 2.934 | **0.003** |
| Yes | 134（20.0） | 122.2±45.53 |  |  |
| No | 536（80.0） | 136.21±50.29 |  |  |

Taking the degree of core information needs as the dependent variable (low, medium, and high were assigned as 1, 2, and 3, respectively), and using urban and rural areas, gender, age, education level, occupation, smoking, and chronic disease as independent variables to conduct multi-factor analysis. Ordinal logistic regression analysis, independent variable assignments are shown in Table 2. The results showed that smoking was the only factor influencing the unmet need for information (P<0.05). Compared with smokers, non-smokers had higher unmet information needs (OR=1.790, 95%CI=1.131-2.838). The multivariate ordinal logistic regression analysis results are shown in Table 3.

**Supplementary 2. Table 2 The assignment method of the influencing factors of the public demand for core information**

| **Item (Independent variable)** | **Assignment** |
| --- | --- |
| **Area** | 1=Urban, 0=Rural |
| **Age(year)** | 1=＜25, 2=26-45, 3=＞45 |
| **Sex** | 1=Male，0=Female |
| **Education** | 1=Elementary school and below, 2=Junior High/Secondary/High School, 3=College/Undergraduate and above |
| **Smoking** | 1=Yes, 0=No |
| **Chronic disease** | 1=Yes, 0=No |
| **Profession** | 1=Medical worker, 0=Non-medical worker |

**Supplementary 3. Table 3 Multivariate ordinal logistic regression analysis of demand for core information**

| **Item** | **β** | **Standard error** | **P** | **OR** | **95%CI** | |
| --- | --- | --- | --- | --- | --- | --- |
|  |  |  |  |  | **Upper** | **Lower** |
| **Area** |  |  |  |  |  |  |
| Urban |  |  |  | 1.00 |  |  |
| Rural | 0.060 | 0.181 | 0.738 | 1.062 | 0.745 | 1.514 |
| **Sex** |  |  |  |  |  |  |
| Male |  |  |  | 1.00 |  |  |
| Female | 0.348 | 0.193 | 0.071 | 1.416 | 0.971 | 2.065 |
| **Age (year)** |  |  |  |  |  |  |
| <26 | -0.167 | 0.227 | 0.461 | 0.846 | 0.542 | 1.320 |
| 26-45 | 0.142 | 0.229 | 0.535 | 1.153 | 0.736 | 1.804 |
| >45 |  |  |  | 1.00 |  |  |
| **Education** |  |  |  |  |  |  |
| Elementary school and below | -0.418 | 0.457 | 0.360 | 0.658 | 0.269 | 1.613 |
| Junior High /Secondary/High School | -0.105 | 0.190 | 0.580 | 0.900 | 0.621 | 1.306 |
| College/Undergraduate and above |  |  |  | 1.00 |  |  |
| **Profession** |  |  |  |  |  |  |
| Medical worker | -0.368 | 0.254 | 0.147 | 0.692 | 0.421 | 1.138 |
| Non-medical worker |  |  |  | 1.00 |  |  |
| **Smoking** |  |  |  |  |  |  |
| Yes |  |  |  | 1.00 |  |  |
| No | 0.583 | 0.235 | **0.013** | 1.790 | 1.131 | 2.838 |
| **Chronic disease** |  |  |  |  |  |  |
| Yes |  |  |  | 1.00 |  |  |
| No | 0.230 | 0.219 | 0.294 | 1.259 | 0.819 | 1.935 |

Microsoft Excel 2019 and SPSS 25.0 were used to process the results of the questionnaire, and count the proportion of different recommendation levels for each item. The expert positive coefficient, authority coefficient and coordination coefficient respectively represent the active degree of experts in filling out the questionnaire, authority and coordination of opinions.The expert positive coefficient is expressed by the filling rate of the first and second rounds of questionnaires, and the positive coefficient ≥75% indicates a higher degree of positiveness. The expert authority coefficient is expressed by the authority coefficient (Cr), and the specific calculation instructions are shown in Table 3. The expert coordination coefficient is expressed by the coefficient of variation (CV). The coefficient of variation is the ratio of the standard deviation (SD) to the average value. CV≤35% indicates that the recommended opinions have a better degree of coordination.

**Supplementary 3. Table 1 Expert authority index description**

| **Expert authority index** | **Description** | **Score** |
| --- | --- | --- |
| Expert authority (Cr) | Cr=(Ca + Cs)/2 | |
| Basis of judging (Ca) | Experience | 1 |
|  | Theoretical analysis | 0.8 |
|  | Know from others | 0.6 |
|  | Intuit | 0.4 |
| Familiarity (Cs) | Very familiar | 1 |
|  | More familiar | 0.8 |
|  | familiar | 0.6 |
|  | Less familiar | 0.4 |
|  | Unfamiliar | 0.2 |

**Supplementary 3. Table 2 Summary of basic characteristic of experts**

| **Characteristic** | **N** | **Percentage（%）** |
| --- | --- | --- |
| **Gender** |  |  |
| Male | 15 | 46.88 |
| Female | 17 | 53.13 |
| **Occupation** |  |  |
| Health communication worker | 8 | 25.00 |
| Clinician | 5 | 15.63 |
| Health communication research | 4 | 12.50 |
| Nurse | 3 | 9.38 |
| Epidemiologist | 2 | 6.25 |
| Pharmacist | 2 | 6.25 |
| Methodology expert | 2 | 6.25 |
| Health economics expert | 2 | 6.25 |
| Government manager | 2 | 6.25 |
| Audience representative | 2 | 6.25 |
| **Professional title** |  |  |
| Senior title | 20 | 62.50 |
| Vice-senior title | 8 | 25.00 |
| Others | 4 | 12.50 |
| **Experience** |  |  |
| ＜1 years | 7 | 21.88 |
| 1-5 years | 7 | 21.88 |
| 6-10 years | 4 | 12.50 |
| 11-20 years | 11 | 34.38 |
| ≥20 years | 3 | 9.38 |
| **Work unit** |  |  |
| Medical institutions | 7 | 21.88 |
| Teaching and Research Unit | 14 | 43.75 |
| Administration Department | 8 | 25.00 |
| Others | 3 | 9.38 |

As shown in Table 5, 32 experts are classified according to their occupation and the number of years they have been engaged in tobacco control work, and the average authority coefficients of experts in different groups are obtained.

**Supplementary 3. Table 3 The average expert authority coefficients in different occupations and different years of working in tobacco control**

| **Characteristic** | **Cr average** | **N=32** |
| --- | --- | --- |
| **Occupation** |  |  |
| Health Communication Worker | 0.68 | 8 |
| Clinician | 0.70 | 5 |
| Health Communication Researcher | 0.63 | 4 |
| Nurse | 0.71 | 3 |
| Epidemiologist | 0.78 | 2 |
| Pharmacist | 0.70 | 2 |
| Methodologist | 0.64 | 2 |
| Health Economics Expert | 0.66 | 2 |
| Government Manager | 0.63 | 2 |
| Audience Representative | 0.57 | 2 |
| **Experience** |  |  |
| ＜1 years | 0.66 | 7 |
| 1-5 years | 0.65 | 7 |
| 6-10 years | 0.69 | 4 |
| 11-20 years | 0.71 | 11 |
| ≥20 years | 0.69 | 3 |

**Supplementary 3. Table 4 The outcome of expert assessment and the degree of expert coordination**

| **Core information** | | **Strength of recommendation** | **Average** | **SD** | **CV** |
| --- | --- | --- | --- | --- | --- |
| **1 Cardiovascular disease in women** | | | | | |
| 1.1 | Coronary Heart Disease（risk） | Appropriate | 4.469 | 0.621 | 0.139 |
| 1.2 | Coronary Heart Disease（mortality） | Appropriate | 4.500 | 0.622 | 0.138 |
| 1.3 | Heart failure（mortality） | Appropriate | 4.469 | 0.621 | 0.139 |
| 1.4 | sudden cardiac death | Appropriate | 4.281 | 0.813 | 0.190 |
| 1.5 | Stroke（risk） | Appropriate | 4.344 | 0.745 | 0.172 |
| 1.6 | Stroke（mortality） | Appropriate | 4.313 | 0.821 | 0.190 |
| 1.7 | Cardiovascular disease（risk） | Appropriate | 4.500 | 0.622 | 0.138 |
| 1.8 | Cardiovascular disease（mortality） | Appropriate | 4.531 | 0.621 | 0.137 |
| **2 diabetes in women** | | | | | |
| 2.1 | Type 2 diabetes | Equivocal | 3.879 | 0.927 | 0.239 |
| **3 Rheumatoid arthritis in women** | | | | | |
| 3.1 | Rheumatoid arthritis | Equivocal | 3.545 | 0.754 | 0.213 |
| 3.2 | Female vs Male | Equivocal | / | / | / |
| 3.3 | Prevalence excluding anthropogenic influences | Inappropriate | / | / | / |
| 3.4 | Rheumatoid factor | Inappropriate | / | / | / |
| 3.5 | Estrogen-related drug use | Inappropriate | / | / | / |
| **4 Respiratory diseases in women** | | | | | |
| 4.1 | Chronic obstructive pulmonary disease | Appropriate | 4.594 | 0.560 | 0.122 |
| **5 Female digestive disorders** | | | | | |
| 5.1 | Pancreatitis（risk） | Equivocal | 3.906 | 0.856 | 0.219 |
| 5.2 | Chronic pancreatitis（risk） | Equivocal | 3.667 | 0.784 | 0.214 |
| 5.3 | Pancreatic cancer（risk） | Equivocal | 3.938 | 0.801 | 0.203 |
| 5.4 | Pancreatic cancer（mortality） | Equivocal | 3.969 | 0.967 | 0.244 |
| 5.5 | Liver Cancer（risk） | Equivocal | 4.000 | 0.842 | 0.211 |
| 5.6 | Liver Cancer（mortality） | Equivocal | 4.037 | 0.706 | 0.175 |
| 5.7 | Stomach Cancer（risk） | Equivocal | 4.037 | 0.808 | 0.200 |
| 5.8 | Upper Gastrointestinal Cancer（risk） | Equivocal | 4.037 | 0.649 | 0.161 |
| 5.9 | Colon cancer（risk） | Equivocal | 3.815 | 0.736 | 0.193 |
| 5.10 | Rectal cancer（risk） | Equivocal | 3.778 | 0.698 | 0.185 |
| 5.11 | Diverticular disease（risk） | Equivocal | 3.667 | 0.679 | 0.185 |
| 5.12 | Colitis（risk） | Equivocal | 3.704 | 0.724 | 0.195 |
| **6 Female mental illness** | | | | | |
| 6.1 | Alzheimer's disease | Equivocal | 3.741 | 1.259 | 0.336 |
| 6.2 | All-cause dementia | Equivocal | 3.704 | 1.171 | 0.316 |
| **7 Non-pregnant female reproductive disorders** | | | | | |
| 7.1 | Endometrial cancer | Equivocal | 3.938 | 0.801 | 0.203 |
| 7.2 | Vaginitis | Equivocal | 3.656 | 0.865 | 0.237 |
| 7.3 | Cervical cancer | Equivocal | 3.844 | 0.920 | 0.239 |
| 7.4 | Breast cancer | Equivocal | 3.969 | 0.822 | 0.207 |
| 7.5 | Ovarian cancer | Equivocal | 3.778 | 0.801 | 0.212 |
| 7.6 | Ovarian Adverse Reactions | Equivocal | 3.656 | 0.937 | 0.256 |
| 7.7 | Abnormal increase in menstrual frequency | Equivocal | 3.875 | 0.793 | 0.205 |
| 7.8 | Early age of spontaneous menopause | Equivocal | 3.969 | 0.822 | 0.207 |
| **8 Pregnant women and their fetuses** | | | | | |
| 8.1 | Adverse effects on all stages of reproduction | Equivocal | 4.094 | 0.818 | 0.200 |
| 8.2 | Abnormal pregnancy | Equivocal | 4.000 | 0.803 | 0.201 |
| 8.3 | Postpartum depression | Equivocal | 3.926 | 0.958 | 0.244 |
| 8.4 | Fetal schizophrenia | Equivocal | 3.889 | 1.050 | 0.270 |
| 8.5 | Fetal attention deficit and hyperactivity disorder | Equivocal | 3.926 | 0.829 | 0.211 |
| 8.6 | Fetal coronary artery disease | Appropriate | 4.500 | 0.568 | 0.126 |

**Supplementary 3. Table 5 Summary of the results of two rounds of expert assessment survey**

| **Core information** | | **The first round of expert assessment survey** | | | | | **The second round of expert assessment survey** | | | | |
| --- | --- | --- | --- | --- | --- | --- | --- | --- | --- | --- | --- |
|  |  | **Consensus** | **Voting ratio (%)** | | | | **Consensus** | **Voting ratio (%)** | | | |
|  |  |  | **“5”** | **“4”** | **“2”** | **“1”** |  | **“5”** | **“4”** | **“2”** | **“1”** |
| **1 Cardiovascular disease in women** | | Appropriate | 71.88 | 25.00 | 0 | 3.13 | Appropriate | 71.88 | 25.00 | 0 | 3.13 |
| 1.1 | Coronary Heart Disease（risk） | Appropriate | 56.25 | 40.63 | 0 | 0 | Appropriate | 56.25 | 40.63 | 0 | 0 |
| 1.2 | Coronary Heart Disease（mortality） | Appropriate | 59.38 | 37.50 | 0 | 0 | Appropriate | 59.38 | 37.50 | 0 | 0 |
| 1.3 | Heart failure（mortality） | Appropriate | 56.25 | 40.63 | 0 | 0 | Appropriate | 56.25 | 40.63 | 0 | 0 |
| 1.4 | sudden cardiac death | Appropriate | 53.13 | 28.13 | 0 | 0 | Appropriate | 53.13 | 28.13 | 0 | 0 |
| 1.5 | Stroke（risk） | Appropriate | 53.13 | 34.38 | 0 | 0 | Appropriate | 53.13 | 34.38 | 0 | 0 |
| 1.6 | Stroke（mortality） | Appropriate | 56.25 | 25.00 | 0 | 0 | Appropriate | 56.25 | 25.00 | 0 | 0 |
| 1.7 | Cardiovascular disease（risk） | Appropriate | 59.38 | 37.50 | 0 | 0 | Appropriate | 59.38 | 37.50 | 0 | 0 |
| 1.8 | Cardiovascular disease（mortality） | Appropriate | 62.50 | 34.38 | 0 | 0 | Appropriate | 62.50 | 34.38 | 0 | 0 |
| **2 diabetes in women** | | Equivocal | 43.75 | 28.13 | 3.13 | 3.13 | Equivocal | 33.33 | 62.96 | 0 | 0 |
| 2.1 | Type 2 diabetes | Equivocal | 31.25 | 34.38 | 6.25 | 0 | Equivocal | 31.25 | 34.38 | 6.25 | 0 |
| **3 Rheumatoid arthritis in women** | | Equivocal | 28.13 | 34.38 | 6.25 | 3.13 | Equivocal | 3.70 | 62.96 | 7.41 | 0 |
| 3.1 | Rheumatoid arthritis | Equivocal | 12.50 | 34.38 | 3.13 | 0 | Equivocal | 12.50 | 34.38 | 3.13 | 0 |
| 3.2 | Female vs Male | Inappropriate | 12.50 | 28.13 | 12.50 | 0 | Equivocal | 0 | 40.74 | 11.11 | 0 |
| 3.3 | Prevalence excluding anthropogenic influences | Inappropriate | 15.63 | 25.00 | 9.38 | 0 | Inappropriate | 3.70 | 37.04 | 7.41 | 0 |
| 3.4 | Rheumatoid factor | Inappropriate | 12.50 | 34.38 | 9.38 | 0 | Inappropriate | 3.70 | 33.33 | 7.41 | 0 |
| 3.5 | Estrogen-related drug use | Inappropriate | 18.75 | 28.13 | 9.38 | 0 | Inappropriate | 3.70 | 44.44 | 0 | 0 |
| **4 Respiratory diseases in women** | | Appropriate | 90.63 | 6.25 | 0 | 3.13 | Appropriate | 90.63 | 6.25 | 0 | 3.13 |
| 4.1 | Chronic obstructive pulmonary disease | Appropriate | 65.63 | 34.38 | 0 | 0 | Appropriate | 65.63 | 34.38 | 0 | 0 |
| **5 Female digestive disorders** | | Equivocal | 31.25 | 46.88 | 3.13 | 0 | Equivocal | 31.25 | 46.88 | 3.13 | 0 |
| 5.1 | Pancreatitis（risk） | Equivocal | 34.38 | 28.13 | 0 | 0 | Equivocal | 34.38 | 28.13 | 0 | 0 |
| 5.2 | Chronic pancreatitis（risk） | Equivocal | 28.13 | 31.25 | 0 | 3.13 | Equivocal | 7.41 | 59.26 | 0 | 3.70 |
| 5.3 | Pancreatic cancer（risk） | Equivocal | 31.25 | 37.50 | 0 | 0 | Equivocal | 31.25 | 37.50 | 0 | 0 |
| 5.4 | Pancreatic cancer（mortality） | Equivocal | 37.50 | 34.38 | 0 | 3.13 | Equivocal | 37.50 | 34.38 | 0 | 3.13 |
| 5.5 | Liver Cancer（risk） | Equivocal | 37.50 | 31.25 | 0 | 0.00 | Equivocal | 37.50 | 31.25 | 0 | 0.00 |
| 5.6 | Liver Cancer（mortality） | Equivocal | 37.50 | 28.13 | 0 | 3.13 | Equivocal | 25.93 | 51.85 | 0 | 0 |
| 5.7 | Gastric Cancer（risk） | Equivocal | 34.38 | 31.25 | 0 | 3.13 | Equivocal | 22.22 | 66.67 | 0 | 3.70 |
| 5.8 | Upper Gastrointestinal Cancer（risk） | Equivocal | 31.25 | 40.63 | 3.13 | 3.13 | Equivocal | 18.52 | 70.37 | 3.70 | 0 |
| 5.9 | Colon cancer（risk） | Equivocal | 28.13 | 37.50 | 3.13 | 3.13 | Equivocal | 14.81 | 55.56 | 3.70 | 0 |
| 5.10 | Rectal cancer（risk） | Equivocal | 31.25 | 31.25 | 3.13 | 3.13 | Equivocal | 11.11 | 59.26 | 3.70 | 0 |
| 5.11 | Diverticular disease（risk） | Equivocal | 15.63 | 34.38 | 6.25 | 3.13 | Equivocal | 7.41 | 55.56 | 3.70 | 0 |
| 5.12 | Colitis（risk） | Equivocal | 18.75 | 37.50 | 3.13 | 3.13 | Equivocal | 3.70 | 70.37 | 0 | 3.70 |
| **6 Female mental illness** | | / | / | / | / | / | Equivocal | 18.52 | 33.33 | 7.41 | 7.41 |
| 6.1 | Alzheimer's disease | / | / | / | / | / | Equivocal | 33.33 | 29.63 | 0 | 11.11 |
| 6.2 | All-cause dementia | / | / | / | / | / | Equivocal | 29.63 | 29.63 | 3.70 | 7.41 |
| **7 Non-pregnant female reproductive disorders** | | Equivocal | 40.63 | 40.63 | 3.13 | 3.13 | Equivocal | 40.63 | 40.63 | 3.13 | 3.13 |
| 7.1 | Endometrial cancer | Equivocal | 31.25 | 37.50 | 0 | 0 | Equivocal | 31.25 | 37.50 | 0 | 0 |
| 7.2 | Cervical cancer | Equivocal | 31.25 | 34.38 | 6.25 | 0 | Equivocal | 31.25 | 34.38 | 6.25 | 0 |
| 7.3 | Breast cancer | Equivocal | 34.38 | 34.38 | 0 | 0 | Equivocal | 34.38 | 34.38 | 0 | 0 |
| 7.4 | Ovarian cancer | / | / | / | / | / | Equivocal | 18.52 | 44.44 | 3.70 | 0 |
| **8 Pregnant women and their fetuses** | | / | / | / | / | / | Appropriate | 74.07 | 25.93 | 0 | 0 |
| 8.1 | Adverse effects on all stages of reproduction | Equivocal | 40.63 | 34.38 | 0 | 0 | Equivocal | 40.63 | 34.38 | 0 | 0 |
| 8.2 | Abnormal pregnancy | Equivocal | 34.38 | 37.50 | 0 | 0 | Equivocal | 34.38 | 37.50 | 0 | 0 |
| 8.3 | Postpartum depression | / | / | / | / | / | Equivocal | 33.33 | 33.33 | 7.41 | 0 |
| 8.4 | Fetal schizophrenia | / | / | / | / | / | Equivocal | 33.33 | 33.33 | 3.70 | 3.70 |
| 8.5 | Fetal attention deficit and hyperactivity disorder | / | / | / | / | / | Equivocal | 25.93 | 44.44 | 3.70 | 0 |
| 8.6 | Fetal coronary artery disease | Appropriate | 56.25 | 43.75 | 0 | 0 | Appropriate | 56.25 | 43.75 | 0 | 0 |
